# Insect herbivory time-course reveals unique temporal transcriptomic responses in resistant and susceptible grapevines

**DOI:** 10.3389/fpls.2026.1874271

**Published:** 2026-08-19

**Authors:** Cullen W. Dixon, Andrea R. Gschwend

**Affiliations:** 1 Department of Horticulture and Crop Science, The Ohio State University, Columbus, OH, United States; 2 Center for Applied Plant Sciences, The Ohio State University, Columbus, OH, United States

**Keywords:** grapevine, insect herbivory resistance, *Popillia japonica*, RNA-seq, systemic response, time-course, *Vitis labrusca*, *Vitis vinifera*

## Abstract

Grapevine (*Vitis*) is a crop of global importance. Insect pests, such as *Popillia japonica* (Japanese beetle), threaten yields of this high value crop. *Vitis labrusca* (accession ‘GREM4’) exhibits the highly-advantageous trait of resistance to Japanese beetles while *Vitis vinifera* is susceptible (cultivar ‘PN40024’). Herein, we undertook an insect herbivory time-course transcriptomic study to identify gene expression differences, and associated processes and pathways, which differ, both temporally and spatially, between ‘GREM4’ and ‘PN40024’ under insect herbivory. Upon herbivory, the number of significantly differentially expressed genes (DEGs) increased over time in ‘GREM4’ but decreased in ‘PN40024’. ‘GREM4’ exhibited greater defense gene enrichment after 4h of herbivory compared to 30min, conversely, ‘PN40024’ exhibited a greater 30min response which diminished by 4h. In systemic leaves, ‘GREM4’ exhibited 10x more DEGs than ‘PN40024’. Across herbivory and systemic responses, genes associated with insect herbivory defense, including flavonoids, phenylpropanoids, acyltransferases, and signaling-pathways, were implicated in greater numbers, or exclusively, in ‘GREM4’ compared to ‘PN40024’ and likely impact insect resistance. Our findings provide insight into the genetic variation, processes, and pathways which underly insect herbivory resistance in *Vitis.* We report candidate genes and biological pathways for future functional studies and breeding initiatives to advance cultivar development and yields.

## 1 Introduction

Insect herbivory is a major stressor that impacts plant health and productivity. Diverse physical and chemical defenses have evolved in plants to dissuade biological stressors, some of which are constitutive while others are inducible. Upon herbivory perception, inducible transcriptomic responses are initiated by protein and hormone signals resulting in metabolic changes *in planta*, including the production of secondary metabolites with insect repellent or insecticidal properties - these include flavonoids, such as 3-deoxyanthocyanidins (Kariyat et al., 2019; Chatterjee et al., 2022), flavan-3-ols (Kumar and Yadav, 2017; Zhang et al., 2022a), and tannins (Chen et al., 2022), phenylpropanoids (He et al., 2019; Dixit et al., 2020; Zhang et al., 2022b, Zhang et al., 2022a)), including cinnamic acid (Wang et al., 2022), and terpenes, such as monoterpenes (Chiu et al., 2017; Giunti et al., 2020; Wang et al., 2023), sesquiterpenes (Quan et al., 2018; Liu et al., 2020; Pavela et al., 2021), and diterpenes (Sun et al., 2022). While this general schema of defense response is generally conserved across plant species, the nuances of the transcriptomic and metabolomic responses often differ, thus influencing defense efficacy (Zhu et al., 2023; Jing et al., 2024).

Timing and duration of responses to insect herbivory are thought to impact defense effectiveness. For example, a time-course transcriptomic analysis of maize (*Zea mays*) under corn leaf aphid (*Rhopalosiphum maidis*) herbivory found that the response altered significantly over time, with the greatest transcriptomic response occurring within the first few hours (4h and 8h) of herbivory (Tzin et al., 2015); Additionally, different sets of genes and metabolites were found in the first 8h of herbivory compared to later timepoints (48h and 96h). Further, transcriptomic alterations can be highly dynamic in the first few hours of insect herbivory. In maize cultivar ‘B73’ under herbivory, only 41% of differentially expressed genes (DEGs) were conserved between the 1h and 4h timepoints, meanwhile, only 38% of DEGs were conserved between 30min and 3h timepoints in rice (*Oryza sativa*) (Tzin et al., 2017; Zhuang et al., 2022). Moreover, both maize cultivar B73 and rice displayed distinct temporal trends for genes in multiple defense pathways, including the jasmonic acid (JA) biosynthesis and phenylpropanoid biosynthesis pathways (Tzin et al., 2015; Zhuang et al., 2022). These findings indicate that temporally-specific gene expression can occur upon insect herbivory, can occur within 4h following herbivory, and can vary between species, illustrating the complex regulatory landscape of gene expression upon insect herbivory. However, only a limited number of studies have explored early (0-4h) *in planta* transcriptomic responses to insect herbivory, resulting in a gap in knowledge of this critical period.

Systemic responses and defensive priming are critical to insect herbivory defense, however, the scope and timing of systemic responses immediately following insect herbivory between resistant and susceptible plants remain largely unexplored. A systemic response is an inducible response which conveys a signal from a local to a distal region of the plant (Zhou et al., 2020). Systemic responses can occur very rapidly (Glauser et al., 2009; Johns et al., 2021), and in some scenarios, are observed in as little as 2min (Toyota et al., 2018). Systemic responses often result in ‘defensive priming’ (priming) – a process wherein prior exposure to a stressor enhances plant defenses, which can be observed in local and distal tissues. For instance, in tea (*Camellia sinensis*) attacked by tea geometrid (*Ectropis obliqua*), DEGs associated with phenylpropanoid biosynthesis, flavonoid biosynthesis, amino-acid metabolism, aromatic compound production, calcium ion signaling, ROS, and phytohormonal regulation were identified in distal, unafflicted leaves, compared to plants without geometrid feeding, suggesting increased defense in such tissues (Zhou et al., 2020). While past studies demonstrate that systemic responses can prime defenses against insect herbivory in unafflicted tissues in diverse species (including rice, maize, husk tomato (*Physalis philadelphica*), and soybean (*Glycine max*)), these studies have relied on mechanical damage combined with pest oral secretions (ul Malook et al., 2019, ul Malook et al., 2021), involved live insect feeding but with sampling intervals spanning days (Dryburgh and Davis, 2021; Meza-Canales et al., 2022), or examined only a single resistant or susceptible genotype without comparing responses (Malhotra et al., 2022; Li et al., 2023b). Previous studies have demonstrated that systemic responses and priming impact insect herbivory, but a deeper understanding of the transcriptomic response and timing following insect herbivory initiation (0-4h) in resistant and susceptible plants is necessary.

Grapes (*Vitis*) have been a part of human diets for centuries and are important agricultural economic drivers (United States Department of Agriculture - National Agricultural Statistics Service, 2023). Therefore, protecting grapevines from yield-reducing stresses is paramount. Insect herbivory can reduce the photosynthetic capability of the canopy, affecting grape yield (Boucher and Pfeiffer, 1989; Pfeiffer, 2012). Japanese beetles (*Popillia japonica*), a polyphagous invasive insect to North America, are major pests of grapevines in the U.S. (Potter and Held, 2002; Mercader and Isaacs, 2003; United States Department of Agriculture, 2015 and USDA-APHIS, 2015; MacGregor et al., 2016). Some grapevine species, such as *Vitis labrusca*, which are native to the northeastern U.S. and southeastern Canada, are highly fit in their local environment and are resistant to many abiotic (Dami, 2007) and biotic (Cadle-Davidson, 2008; Gee et al., 2008; Nascimento-Gavioli et al., 2019) stressors, including Japanese beetles (Dixon and Gschwend, 2024). Meanwhile, *Vitis vinifera* varieties, which are widely cultivated for their excellent flavor, are susceptible to many insects and pathogens (Dami et al., 2005; Smith, 2005; Cadle-Davidson, 2008; Nascimento-Gavioli et al., 2019). A previous study found *Vitis labrusca* acc. ‘GREM4’ was resistant to Japanese beetle herbivory, while *Vitis vinifera* cv. ‘PN40024’ was susceptible (Dixon and Gschwend, 2024). In the study, ‘GREM4’ exhibited significant up-regulation of genes implicated in terpene biosynthesis, flavonoids biosynthesis, and plant-pathogen interactions compared to ‘PN40024’. ‘GREM4’ also exhibited greater constitutive expression of genes related to secondary metabolite biosynthesis and plant-pathogen interaction compared to ‘PN40024’. In another study, comparative genomic analyses of ‘GREM4’ and ‘PN40024’ revealed unique genomic features, such as structural variations, indels, and single nucleotide polymorphisms, that were linked to heightened resistance to abiotic and biotic stresses (Li and Gschwend, 2023).

Despite this body of work, an important question remains – Are there differences in the temporal and spatial transcriptomic responses in the first four hours following insect herbivory initiation between resistant and susceptible grapevines? Currently, few studies across plant life have investigated the differences in temporal transcriptomic responses between resistant and susceptible accessions, let alone species, to insect herbivory, and, in studies which do, they span large timescales, ranging from many hours to days (Broekgaarden et al., 2007; Wang et al., 2014; Li et al., 2016; Tetreault et al., 2019; Dixit et al., 2020). Herein, we performed a comparative time-course study to investigate the direct and systemic transcriptomic responses of resistant (GREM4’) and susceptible (‘PN40024’) grapevines to insect herbivory to identify temporal and spatial alterations in gene expression after 30min, 1h, and 4h of feeding and identify genes and pathways contributing to the response.

## 2 Materials and methods

### 2.1 Herbivory time-course study

**Figure 1:**
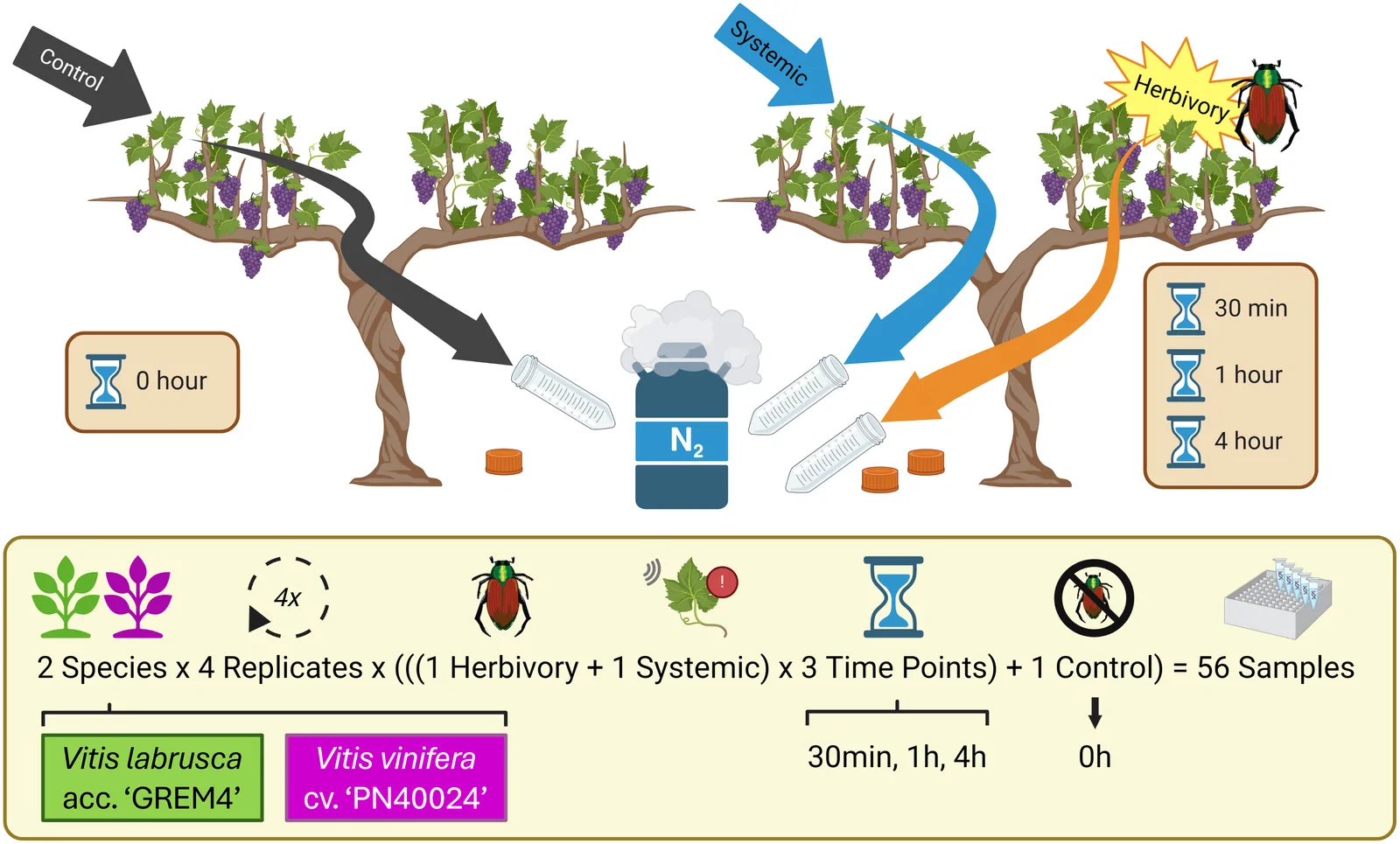
A depiction of the experimental design of the comparative time-course transcriptomic study. 0h samples were collected from a separate grapevine plant, to avoid confounding transcriptomic responses due to leaf removal. Meanwhile, herbivory and systemic samples were collected from the same plant concurrently after 30min, 1h, and 4h of beetle herbivory.

Transparent mesh bags containing one semi-starved adult Japanese beetle were applied to mature leaves of two to three-year-old *Vitis labrusca* acc. ‘GREM4’ (PI-588583) and *Vitis vinifera* cv. ‘PN40024’ (DVIT-908) plants (**Supplementary Figure 1A**). ‘GREM4’ and ‘PN40024’ were selected due to the availability of reference genomes (Li and Gschwend, 2023) and their variation in insect herbivory resistance (Dixon and Gschwend, 2024). Beetles fed for 30min, 1h, or 4h, and timing was closely monitored, beginning once a hole from feeding was visible in the leaf (**Figure 1**; **Supplementary Figure 1B**) – these were ‘herbivory’ leaves. The herbivory timespans were selected based on the time it takes for transcriptional and metabolite alterations to occur *in planta* based on previous studies (Köllner et al., 2010; Pandey et al., 2017). Leaf area damaged per leaf was consistent within species × tissue × timepoint groups, but was significantly greater in ‘PN40024’ than in ‘GREM4’ (Dixon and Gschwend, 2024). On average, the Japanese beetles ate 0.49, 7.20, and 3.86 mm^2^ of the GREM4 leaves at 30min, 1h, and 4h, respectively, and ate 17.47, 33.03, and 32.28 mm^2^ of the PN40024 leaves at 30min, 1h, and 4h, respectively (Dixon and Gschwend, 2024).

‘Systemic’ leaves were also enclosed in a mesh bag, but without a beetle, and were applied simultaneously with placement of herbivory bags and beetles. Herbivory and systemic leaves were collected from the same plant, but separate canes (**Figure 1**). ‘Control’ leaves (0h), which did not experience any herbivory, were collected once feeding was noted on an herbivory leaf (thus 0h), but from a separate clonally propagated plant to prevent confounding transcriptomic effects on the herbivory and systemic leaves precipitated by leaf removal.

Leaves from all groups were of the same maturity, size, and cane position but were otherwise chosen at random beyond these parameters. The greenhouse space was broken into nine spatially equally sized sectors in a 3x3 formation with three to four plants per sector. Plants for experimentation were chosen by selecting one plant at random from within a sector, until all sectors were used, at which point, a sector would become re-used but a different plant selected. On any given experimentation day, all sectors were rarely used. Plants selected for experimentation were subjected to a “washout period” of at least four days to minimize potential transcriptomic artifacts carried over from the feeding run.

Following each herbivory treatment, the ‘herbivory’, ‘systemic’, and ‘control’ leaves were rapidly photographed (**Supplementary Figure 1B**), then immediately placed in 50mL conical tubes, flash-frozen in liquid nitrogen, and stored at -80 °C until RNA isolation for RNA-sequencing. In total, 56 samples (four herbivory and four systemic biological replicates for each of three timepoints, plus four biological replicates of 0h samples, for both species) were collected. Despite having four biological replicates per species x tissue x treatment combination, given the multi-factorial design of the experiment, some real, but subtle, expression changes may not have been detected given the sample size used.

Additional details about plant material selection and growth, experimental design, and insect collection can be found in Dixon and Gschwend, 2024, which originated the data set utilized in this study, and on GitHub (https://github.com/cdixo/Inter-species-and-Herbivory-Publication.git).

### 2.2 RNA isolation and sequencing

A Sigma-Aldrich Spectrum Plant Total RNA Kit was used to isolate RNA from the leaf samples, following the manufacturer’s protocol, in seven batches. RNA quality and quantity were assessed and standardized across samples via Qubit, Nanodrop, Bioanalyzer, and formaldehyde gel. Library preparation and Illumina NovaSeq 6000 paired-end RNA sequencing (150bp, 20M reads per sample) were performed by Novogene in a single batch (with multiplexing). Raw reads were cleaned using Trimmomatic (Bolger et al., 2014) with options set as ‘PE -phred33 ILLUMINACLIP: Novogene-PE.fa:2:30:10 SLIDINGWINDOW:4:5 LEADING:5 TRAILING:5 MINLEN:36’. Sequencing results were reviewed via PCA and Pearson correlation hierarchical clustering and no batch effects were identified. Samples generally clustered via biological variables such as species, tissue, and timepoint.

STAR (Dobin et al., 2013) was used to align the ‘GREM4’ and ‘PN40024’ RNA-seq reads to their respective genomes: ‘GREM4’ (*Vitis labrusca* acc. ‘GREM4’ genome and gene annotation (Li and Gschwend, 2023; Dixon and Gschwend, 2024)) and ‘PN40024’ (*Vitis vinifera* cv. ‘PN40024’ 12x.2 genome (Canaguier et al., 2017) and v4.56 annotation (The Institut National de la Recherche Agronomique, 2022; Velt et al., 2023; Ensembl Plants release 58 and EMBL-EBI, 2024)). The ‘GREM4’ genome possesses 37,443 annotated genes, whereas the ‘PN40024’ genome has 35,133 annotated genes. 82.41% and 90.27% of the reads aligned in ‘GREM4’ and ‘PN40024’, respectively. 10.50% and 5.78% of aligned reads were multi-mapping in ‘GREM4’ and ‘PN40024’, respectively, therefore, to generate a count matrix which better accounted for multi-mapping reads, the program CoCo (Deschamps-Francoeur et al., 2019) was implemented using function ‘coco correct_counts’. Significantly up- and down-regulated differentially expressed genes (DEGs), defined as p.adj ≤ 0.05 and |log2FoldChange| ≥ 2, were ascertained by comparing herbivory or systemic leaf transcriptomes at each individual timepoint (30min, 1h, and 4h) to 0h controls via DESeq2 (Love et al., 2014) (e.g. – ‘GREM4’ 30min Systemic compared to ‘GREM4’ 0h Control). Volcano plots were generated via R package ‘EnhancedVolcano’ (Blighe et al., 2024). Herbivory and systemic responses were compared via overlap analysis to identify DEGs conserved or unique between herbivory compared to 0h and systemic compared to 0h comparisons (comparisons of lists of DEGs). Venn diagram creation and parsing of DEGs conserved or unique between comparisons were determined via BioInfoRx (BioInfoRx, 2023), BioVenn (Hulsen et al., 2008), Upset (Lex et al., 2014), and molbiotools (Molbiotools, 2023).

Over-Representation Analysis (ORA) was used to identify Gene Ontology (GO) term (Ashburner et al., 2000; Consortium, 2021) enrichment in sets of DEGs via ‘enricher’ (clusterProfiler (Wu et al., 2021)) with a *post-hoc* ‘gsfilter’ (DOSE (Yu et al., 2015)) wherein enrichments with <5 implicated DEGs were removed. Pathway enrichment of DEGs was performed using Kyoto Encyclopedia of Genes and Genomes (KEGG (Kanehisa and Goto, 2000)) via the KEGG Orthology-Based Annotation System-intelligent (KOBAS-i (Bu et al., 2021)), Fisher’s Exact Test statistical analysis method option, wherein enrichments with <5 implicated DEGs were removed. Significance was determined by p.adj ≤ 0.05, and FDR correction was implemented using the Benjamini-Hochberg method, for both analyses.

### 2.3 Homologous gene identification

Orthologous, paralogous, and genome specific genes in the ‘GREM4’ and ‘PN40024’ genomes were identified using OrthoFinder V2.2.5 (Emms and Kelly, 2019), DIAMOND (Buchfink et al., 2021), and custom scripts to detect homology between genes, with manual curation on randomly selected subsets of results via NCBI BLAST (Agarwala et al., 2016) to ensure accuracy. Orthologs were defined as genes that had a one-to-one match in the ‘GREM4’ and ‘PN40024’ genomes, paralogs were additional gene copies of an ortholog present in one genome but not the other, while genome-specific genes did not have a corresponding ortholog in the other species’ genome nor a paralog within the same species. Additional details on the methodology used to identify gene orthology can be found in Dixon and Gschwend, 2024 and on GitHub (https://github.com/cdixo/Inter-species-and-Herbivory-Publication.git).

### 2.4 Data availability

The RNA-seq data used in this study was first generated by Dixon and Gschwend (2024) and can be found at NCBI BioProject ‘PRJNA1070606’ (https://www.ncbi.nlm.nih.gov/bioproject/PRJNA1070606). Additional details of plant material selection and growth, experimental design, insect collection, RNA isolation, RNA sequencing, transcriptomic analysis, and gene homology across genomes can be found in the Dixon and Gschwend (2024) and on GitHub (https://github.com/cdixo/Inter-species-and-Herbivory-Publication.git).

## 3 Results

To investigate the temporal transcriptome responses contributing to insect herbivory resistance in ‘GREM4’, we performed a comparative time-course transcriptomic analysis of ‘GREM4’ and ‘PN40024’ leaves. Samples included: 1) Leaves from plants that did not undergo insect herbivory (0h – control), 2) Leaves subjected to direct Japanese beetle feeding (herbivory), and 3) Leaves from the same plant undergoing herbivory, but from a different cane that was not subjected to direct herbivory (systemic) (**Figure 1**). Herbivory and systemic samples were collected at 30min, 1h, and 4h post-feeding initiation, with four replicates per species, treatment, and timepoint. Four replicates of 0h controls were collected per species, as well. Differential transcriptomic responses within and between ‘GREM4’ and ‘PN40024’ upon Japanese beetle herbivory over a 4h period were determined.

### 3.1 Insect herbivory response

Significantly differentially expressed genes (DEGs) in response to Japanese beetle herbivory were identified in ‘GREM4’ and ‘PN40024’ at 30min, 1h, and 4h by comparing each timepoint to the 0h control (**Supplementary Table 1**), providing insights into the temporal dynamics of transcriptomic responses in resistant ‘GREM4’ and susceptible ‘PN40024’ grapevines.

#### 3.1.1 DEG identification

**Figure 2:**
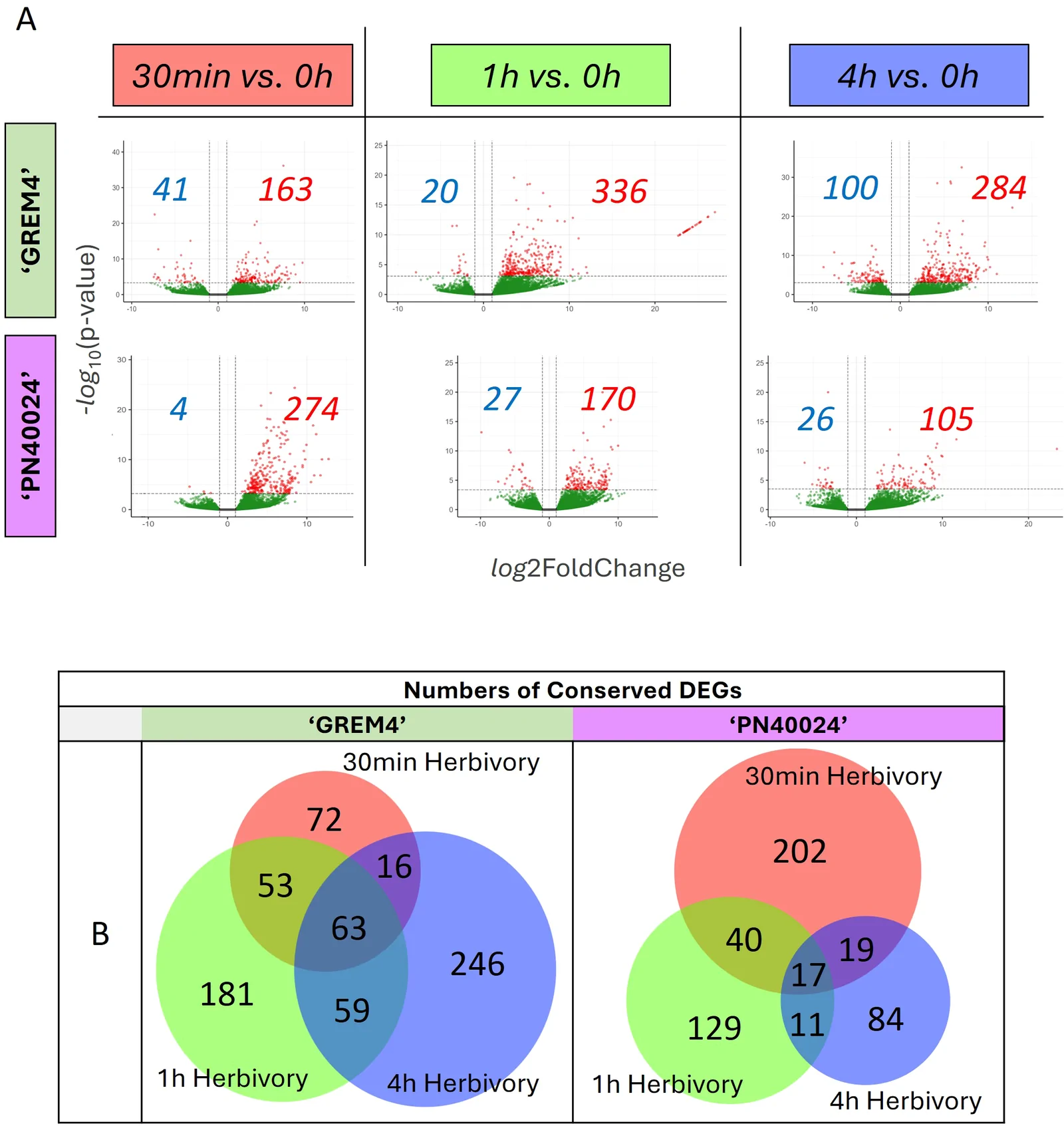
Gene expression results from herbivory leaves after 30min, 1h, and 4h of Japanese beetle herbivory. **(A)** Volcano plots depicting numbers of DEGs in ‘GREM4’ and ‘PN40024’ under insect herbivory when compared to 0h using an |log2FoldChange| ≥ 2 and a p.adj value ≤ 0.05 to determine significance; Grey dashed lines demarcate these thresholds in the plots. Red dots represent genes with an |log2FoldChange| value ≥ 2 and a p.adj value of ≤ 0.05. Green dots represent genes with an |log2FoldChange| value ≥ 2 and a p.adj value of > 0.05. Grey dots represent genes with an |log2FoldChange| value < 2 and a p.adj value of > 0.05. Red and blue inlaid numbers report the numbers of up- and down-regulated DEGs, respectively. **(B)** Venn diagrams depicting the number of overlapping and unique DEGs between 30min, 1h, and 4h of insect herbivory in ‘GREM4’ and ‘PN40024’. Up- and down-regulated DEGs at each timepoint were combined.

In both species, more genes were up-regulated than down-regulated at all timepoints (**Figure 2A**). In ‘GREM4’, the total number of DEGs increased over time from 204 at 30min to 384 at 4h, whereas in ‘PN40024’, DEGs decreased from 278 at 30min to 131 at 4h. These results report an immediate but waning response in ‘PN40024’ contrasted with a progressively increasing response in ‘GREM4’.

#### 3.1.2 DEG conservation

Temporal dynamics of transcriptomic responses are crucial for effective plant defense. Comparing early and late transcriptomic responses between resistant and susceptible species can reveal adaptations enhancing insect herbivory resistance. Accordingly, DEGs conserved and unique to timepoints were next identified. In ‘GREM4’, 690 non-redundant DEGs (549 up-regulated, 141 down-regulated) were identified, with 63 (9%) conserved across all timepoints and 191 (28%) conserved between at least two (**Figure 2B**; **Supplementary Figures 2A**, **3A**). DEGs unique to 30min, 1h, and 4h timepoints accounted for 35%, 51%, and 64% of DEGs at each timepoint, respectively, reporting that while the majority of DEGs at 30min were also expressed at later timepoints, DEGs identified at 1h and 4h were moreso unique to their timepoints. Conserved DEGs in ‘GREM4’ across the three timepoints included genes associated with phenylpropanoids (*VlPAL1-4*), flavonoids (*VlFLS2* and *VlFLS/F3H*), terpenes (*VlTPS14–1* and *VlTPS14-2*), phytohormones (*VlAOS1*, *VlWRKY48*, *VlABR1-like*, *VlERF110*, and *VlACO3*), and senescence (*VlSRG1-1*) (**Supplementary Table 2**).

Comparatively, in ‘PN40024’, 502 non-redundant DEGs (447 up-regulated, 55 down-regulated) were identified, with only 17 (3%) conserved across all timepoints and 87 (17%) conserved between at least two timepoints (**Figure 2B**; **Supplementary Figures 2B**, **3B**; **Supplementary Table 2**). Unique DEGs at 30min, 1h, and 4h accounted for 73%, 65%, and 64% of DEGs at each timepoint, respectively, indicating more distinct temporal gene expression compared to ‘GREM4’ early in herbivory. Conserved DEGs in ‘PN40024’ were associated with ethylene (ETH) (*VvERF073*), JA (*VvTIFY5A*, *VvTIFY5A/JAZ8*, and *VvCYP94C1*), disease resistance (*VvGLIP2-2*), terpene biosynthesis (*VvTPS1-1*), transport (*VvABCG40–2* and *VvER body-like protein*), and development (*VvIKU2*) (**Supplementary Table 2**). Conserved DEGs in ‘PN40024’ were distributed across a broader range of biological pathways, with fewer DEGs identified per pathway, whereas, in ‘GREM4’, fewer pathways were identified but contained greater numbers of DEGs. Overall, ‘GREM4’ exhibited greater conservation of DEGs across timepoints in response to insect herbivory compared to ‘PN40024’. However, in both species, the majority of DEGs were only detected at one timepoint, reporting specific temporal responses.

#### 3.1.3 Top 20 DEGs

To elucidate temporal gene expression dynamics in herbivory leaves upon insect herbivory, we compared the top 20 DEGs, ranked by |log2FoldChange|, at each timepoint between the two species (**Supplementary Table 3**). At 30min in ‘GREM4’, most of the top 20 DEGs were related to phytohormone signaling (*VlSAMT2*, *VlABR1-like*, *VlWIN1*, *VlUMAMITI14/WAT1-related*, *VlHVA22F*, *VlTET8*, and *VlLOG7*) and defense (*VlSPCL-like 45*, *VlCRK26*, *VlUMAMITI14/WAT1-related*, *VlHVA22F*, and *VlBAG6*) while one terpene-related gene was identified (*VlTPS14-1*). Most of the top 20 DEGs in ‘GREM4’ were conserved between other timepoints, though some, like *VlTET8* and *VlHVA22F*, were only significantly differentially expressed at 30min, and were not identified as DEGs in ‘PN40024’. Only three of the top 20 DEGs were down-regulated; these genes were related to water use efficiency, stress tolerance, and photosynthesis. In contrast, top DEGs at 30min in ‘PN40024’ were primarily associated with development (*VvEXO-1*, *VvOPR11-1*, *VvSMR4*, *VvERF109-like 1*, *VvOPR11-2*, and *VvEXO-2*), abiotic stress response (*VvDREB1E-like*, *VvOPR11-1*, *VvERF109-like 1*, *VvAVT1I-1*, *VvERF109-like 2*, and *VvOPR11-2*), or pathogen defense (*VvERF017-1*, *VvERF017-2*, *VvERF109-like 1*, *VvERF017-like*, and *VvERF109-like 2*). Minimal conservation between the top 20 DEGs at 30min in ‘PN40024’ and DEGs in ‘GREM4’ was observed, suggesting distinct transcriptomic responses. Notably, *VvJAZ8* (#12) and *VvTIFY5A/JAZ8* (#6) were two of the top DEGs in ‘PN40024’ at 30min that impact insect defense regulation, generalized defense, wounding response, and JA modulation. In ‘PN40024’, *JAZ8* was significantly differentially expressed at 30min, suggesting an early role in insect herbivory response, meanwhile in ‘GREM4’ it was significantly differentially expressed at a later timepoint (4h). *TIFY5A/JAZ8* was detected as a top DEG in ‘PN40024’ and ‘GREM4’ across timepoints, underscoring its critical role in defense response and JA regulation across time in both species.

At 1h, all top 20 DEGs in ‘GREM4’ were highly up-regulated (23.4-26.2), and were predominantly involved in pathogen defense (*VlPRP4*, *VlBLGU16*, *VlGDSL1-like*, and *VlGLIP1*), cell wall biogenesis (*VlGRP5-like 1*, *VlGRP5-like 2*, *VlPMEI25*, and *VlGUN11*), phytohormones (*VlAOS3*, *VlCYP-like*, and *VlAAE6*), and terpene biosynthesis (*VlBAS isoform X2/CAMS1*, and *VlCYP716A1*). Though many of these top 20 genes had high log2FoldChange values at other timepoints, the p-adjust values at these timepoints did not meet criteria for these genes to be considered DEGs (**Supplementary Tables 1**, **3**). These genes likely play important roles in initiation or progression of defense responses to insect herbivory in ‘GREM4’. In contrast, top DEGs in ‘PN40024’ at 1h had lower log2FoldChange values (8.1-10.0), and only one DEG was down-regulated. These DEGs were predominantly linked to reproduction and development (*VvCTPS1*, *VvSBT1.1*, *VvFLA*, *VvBGAL3*, *VvT2P4.23*, *VvIPUT1*, and *VvABCG6*), phytohormones (*VvIPT*, *VvPME53*, and *VvSAMT3*) and pathogen resistance (*VvIPT* and *VvBGAL3*). Only one of the top 20 DEGs in ‘PN40024’ at 1h was detected as DEGs in ‘GREM4’ at any timepoint *- TIFY5A/JAZ8*.

At 4h, top DEGs in ‘GREM4’ were primarily related to stilbene biosynthesis (*VlSTS1-4*, *VlSTS1-5*, *VlSTS1-3*, *VlSTS1-6*, and *VlSTS1-7*), phytohormones and signaling (*VlSAMT2*, *VlABTR1-like*, *VlGLIP2-1*, *VlTIFY5A/JAZ*, and *VlbHLH167*) and pathogen defense genes (*VlGLIP2–1* and *VlCEP9*). Of note, *VlTPS14-1*, *VlPAL1-1*, and *VlCEP9* were up-regulated at 4h. *TPS14–1* and *PAL1–1* play roles in terpene and phenylpropanoid biosynthesis, respectively, and *CEP9* impacts extracellular signaling, biotic stress responses, and nitrogen management. *OsCEP9* was also up-regulated at ~4h post-bacterial-infection in rice (Mondal et al., 2022), suggesting *CEP9* may serve as a master-regulator of late (4h) biotic stress responses. In ‘PN40024’, top DEGs at 4h were predominantly implicated in growth and development (*VvT2P4.23*, *VvPLA2-ALPHA*, *VvENOD2-1*, *VvENOD2-2*, *VvENOD2-3*, *VvCFP*, and *VvERF1B*) and phytohormones (*VvABR1-like*, *VvTIFY5A/JAZ8*, *VvMYB78*, *VvGH3.1-2/BRU6*, and *VvGH3.1-3*). *ABR1-like* ranked second or third at 30min and 4h in ‘GREM4’ and ‘PN40024’, reporting a consistently high (9.0-11.7) level of expression in both species. *Vitvi01g01801* similarly was a DEG in ‘PN40024’ at only 30min and 4h (17^th^ and 4^th^) but was only a DEG in ‘GREM4’ (104^th^) at 4h. *Vitvi10g00826* was the only top DEG in ‘PN40024’ to be a top DEG across all timepoints.

Overall, the top 20 DEGs at each time point in both species displayed functions related to pathogen response, generalized defense, and phytohormones. However, ‘PN40024’ exhibited significant up-regulation of more growth-related genes, meanwhile greater numbers of genes related to signaling and secondary metabolite biosynthesis were identified in ‘GREM4’, especially at later timepoints. Of 45 top DEGs in ‘GREM4’ that shared an ortholog with ‘PN40024’, only three were a top DEG in ‘PN40024’, at any timepoint, emphasizing the distinct transcriptomic responses between the two species.

#### 3.1.4 Enrichment analyses

**Figure 3:**
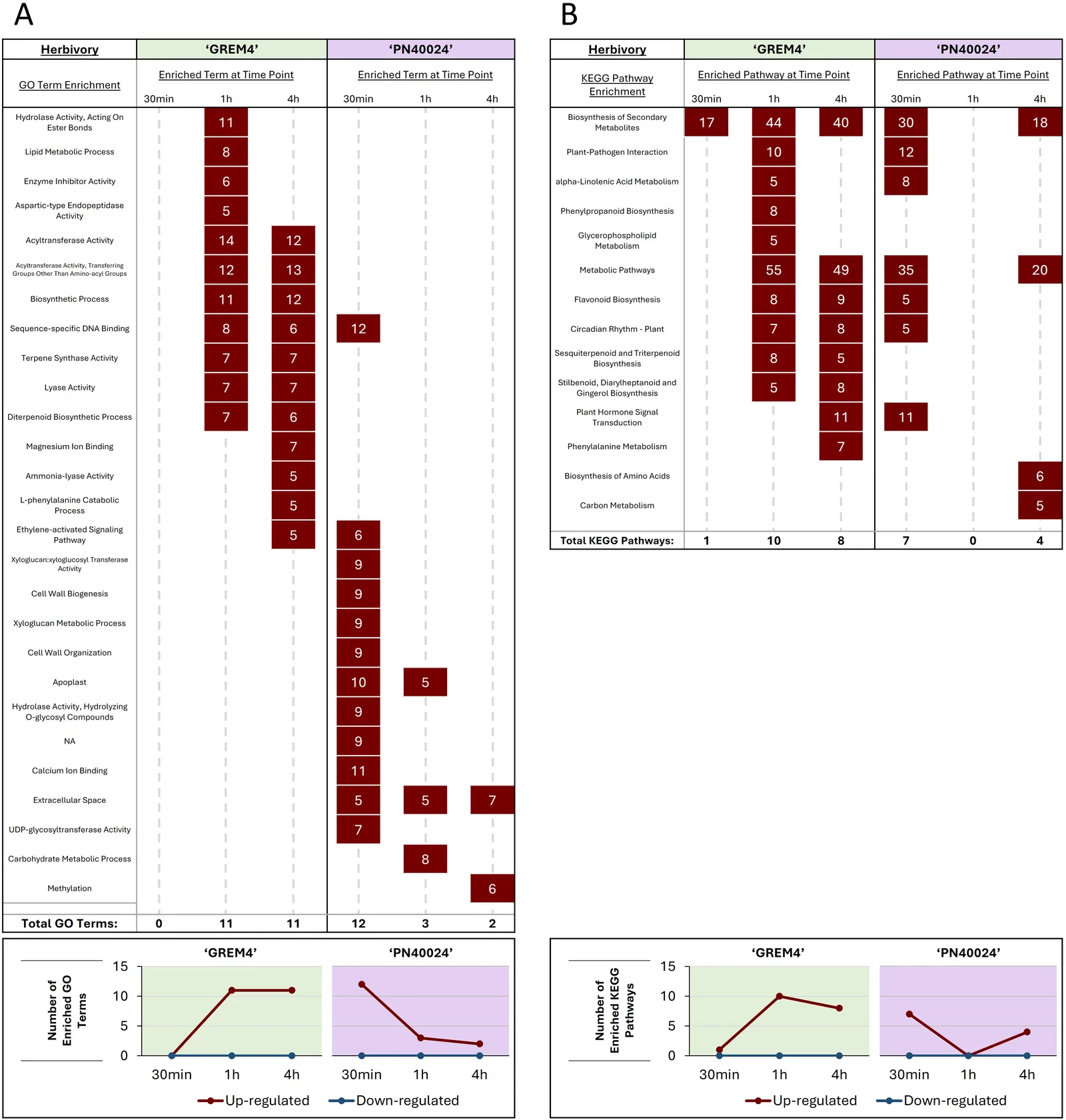
Herbivory DEG GO term **(A)** and KEGG pathway **(B)** enrichments identified for ‘GREM4’ and ‘PN40024’ at 30min, 1h, and 4h. A box to the right of the enrichment category indicates a significant enrichment at the corresponding timepoint and the number in the box indicates the number of DEGs implicated in the biological function or pathway. The color of the box signifies if the DEGs enriched for the function or pathway were up- (maroon) or down-regulated (navy). At the base of both graphics, two line charts graphically illustrate the numbers of enrichments identified in ‘GREM4’ and ‘PN40024’, at each timepoint. The red lines represent the number of enrichments of up-regulated DEGs and blue lines represent the number of enrichments of down-regulated DEGs. Significant enrichments for both GO Terms and KEGG were defined as p.adj ≤ 0.05 and ≥ 5 DEGs.

Over-representation analysis (ORA) identified significantly enriched Gene Ontology (GO) terms (Ashburner et al., 2000; Consortium, 2021) (biological functions) for DEGs at each herbivory timepoint. For the down-regulated DEGs, which varied from 20 to 100 DEGs in ‘GREM4’ and four to 27 DEGs in ‘PN40024’ (**Table 1**; **Figure 2A**), no significant functional enrichments were identified in either species at any timepoint (**Figure 3A**; **Supplementary Table 4**), although some of the most down-regulated genes in ‘GREM4’ included *VlSBTI1.1*, *VlBAG6*, and *VlFTSH6* (**Supplementary Table 3**). Manual review of the functions/putative functions of down-regulated genes revealed a great diversity of functions, therefore, we hypothesize that the general lack of function enrichment in the down-regulated gene sets was due to a combination of the diversity of gene functions and the lack of overall DEGs in the gene sets. Functional enrichments were identified for up-regulated DEGs, which ranged from 163 to 336 DEGs in ‘GREM4’ and 105 to 274 DEGs in ‘PN40024’ in herbivory leaves (**Table 1**; **Figure 2A**). In ‘GREM4’, at 30min, no significant functional enrichments were detected, but at 1h and 4h, DEGs were enriched for 11 functional categories, including acyltransferase activity, secondary metabolite biosynthesis, and hormone signaling (**Figure 3A**; **Supplementary Table 4**). ‘PN40024’ showed an opposite trend, with DEGs enriched for 12 functional categories at 30min, including those related to cell wall biogenesis and cell signaling, but DEGs were only enriched for three functional categories at 1h related to extracellular space and carbohydrate metabolism and two at 4h related to methylation and extracellular space. Notably, seven of the 15 total functional enrichments in ‘GREM4’ were conserved between 1h and 4h, while only two of 14 functional enrichments in ‘PN40024’ persisted between at least two timepoints, suggesting the response in ‘GREM4’ exhibited greater consistency. Between the two species, only two of 26 enriched functional categories were shared (‘sequence-specific DNA binding’ and ‘ethylene-activated signaling pathway’) emphasizing their distinct transcriptional responses.

**Table 1:** Number of herbivory and systemic response DEGs by timepoint and tissue.

			30min	1h	4h
‘GREM4’	Herbivory	Up	163	336	284
		Down	41	20	100
		Total	204	356	384
	Systemic	Up	264	419	131
		Down	127	41	138
		Total	391	460	269
‘PN40024’	Herbivory	Up	274	170	105
		Down	4	27	26
		Total	278	197	131
	Systemic	Up	37	64	8
		Down	2	2	3
		Total	39	66	11

KEGG (Kyoto Encyclopedia of Genes and Genomes (Kanehisa and Goto, 2000)) pathway enrichment analysis identified biological pathways significantly enriched for DEGs. Consistent with ORA findings, no pathways were significantly enriched for down-regulated DEGs, while more pathways were enriched for up-regulated DEGs at 1h (10 pathways) and 4h (8 pathways) in ‘GREM4’, while more were enriched at 30min (7 pathways) in ‘PN40024’ (**Figure 3B**; **Supplementary Table 5**). As noted above, the absence of significant enrichment among down-regulated gene sets, is likely attributable to the functional diversity of the genes and the limited number of genes identified. ‘Biosynthesis of secondary metabolites’ and ‘flavonoid biosynthesis’ were enriched in up-regulated DEGs in both species, but ‘GREM4’ up-regulated DEGs were enriched for additional secondary metabolite pathways at 1h and 4h, including ‘sesquiterpene and triterpene biosynthesis’, ‘phenylpropanoid biosynthesis’, and ‘stilbenoid, diarylheptanoid and gingerol biosynthesis’, which are important to insect herbivory defense. ‘Carbon metabolism’ and ‘biosynthesis of amino acids’ were exclusively enriched for ‘PN40024’ 4h DEGs.

Overall, these data illustrate a later, but more robust and sustained defensive transcriptomic response in ‘GREM4’, particularly at 1h and 4h. While ‘PN40024’ exhibited a stronger early response, ‘GREM4’ exhibited greater activation of key defensive genes and pathways over time, such as secondary metabolite biosynthesis pathways, underscoring the importance of temporal dynamics in herbivory resistance.

### 3.2 Systemic response to insect herbivory

Systemic responses, inducible defense mechanisms triggered by stressors like insect herbivory, transmit signals to unafflicted tissues, enhancing defense (Zhou et al., 2020). To assess their role in grapevine defense, transcriptomic responses in systemic leaves at 30min, 1h, and 4h were compared to control (0h) in both ‘GREM4’ and ‘PN40024’. Systemic leaves were collected from a separate cane of the same plant as the herbivory-afflicted leaves to capture the systemic response (**Figure 1**).

#### 3.2.1 DEG identification

**Figure 4:**
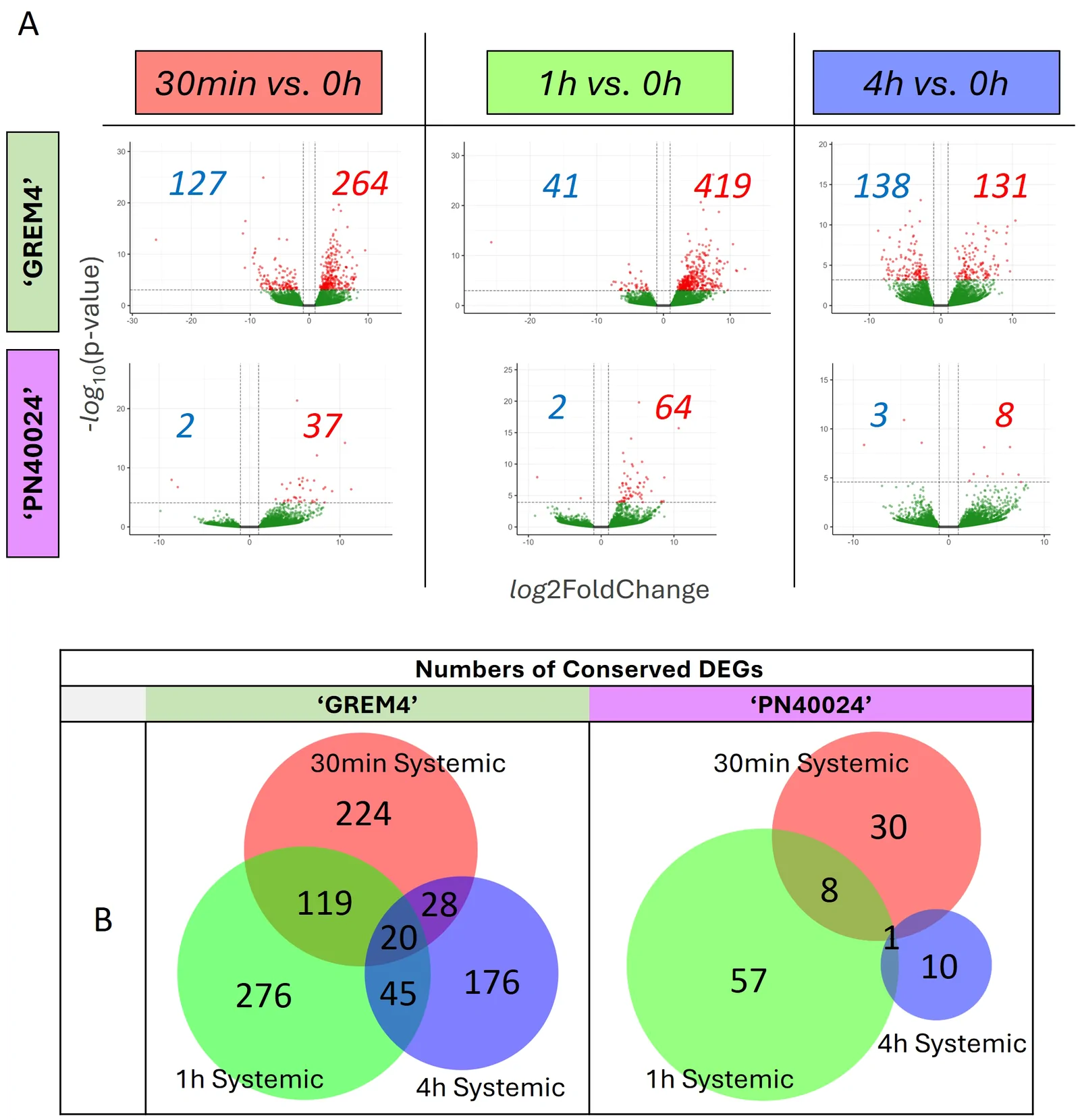
Gene expression results from systemic leaves after 30min, 1h, and 4h of Japanese beetle herbivory. **(A)** Volcano plots depicting numbers of DEGs in ‘GREM4’ and ‘PN40024’ in systemic leaves when compared to 0h using an |log2FoldChange| ≥ 2 and a p.adj value ≤ 0.05 to determine significance; Grey dashed lines demarcate these thresholds in the plots. Red dots represent genes with an |log2FoldChange| value ≥ 2 and a p.adj value of ≤ 0.05. Green dots represent genes with an |log2FoldChange| value ≥ 2 and a p.adj value of > 0.05. Grey dots represent genes with an |log2FoldChange| value < 2 and a p.adj value of > 0.05. Red and blue inlaid numbers report the numbers of up- and down-regulated DEGs, respectively. **(B)** Venn diagrams depicting the number of overlapping and unique DEGs in systemic leaves between 30min, 1h, and 4h of insect herbivory in ‘GREM4’ and ‘PN40024’. Up- and down-regulated DEGs at each timepoint were combined for these Venn diagrams.

In systemic leaves, the majority of DEGs in both species were up-regulated at each timepoint, aside from ‘GREM4’ at 4h wherein up- and down-regulated DEGs were roughly equivalent (**Figure 4A**). ‘GREM4’ systemic leaves had a greater transcriptomic response, with 1,120 total DEGs (888 non-duplicated across timepoints) across all timepoints compared to only 116 (106 non-redundant) in ‘PN40024’. Both ‘GREM4’ and ‘PN40024’ systemic leaves had an early transcriptomic response at 30min (391 and 39 DEGs, respectively), with the greatest number of DEGs in both accessions observed at 1h (460 and 66 DEGs) and the lowest at 4h (269 and 11), however, ‘GREM4’ had a far greater transcriptomic response overall.

#### 3.2.2 DEG conservation

Systemic DEGs conserved across or unique to timepoints were next identified. In ‘GREM4’, 20 (2%) of 888 total non-redundant DEGs were conserved between all timepoints while 212 (24%) were identified between at least two (**Figure 4B**; **Supplementary Figures 2C**, **3A**; **Supplementary Table 2**). Conserved DEGs in ‘GREM4’ were implicated in phenylpropanoid, flavonoid, and terpene biosynthesis, JA and ETH signaling, along with other pathways. Comparatively, in ‘PN40024’, only one of 106 total non-redundant DEGs was conserved between all timepoints, *VvCCR1-like*, while nine DEGs were conserved between at least two (**Figure 4B**; **Supplementary Figures 2D**, **3B**; **Supplementary Table 2**). Most systemic response DEGs were timepoint-specific in both species.

#### 3.2.3 Evolutionary considerations

When apprising the systemic response against a backdrop of evolutionary dynamics, of 888 non-redundant total DEGs in ‘GREM4’, 763 were orthologous to ‘PN40024’ genes, yet only 43 (4.8%) were identified as DEGs in the systemic response of ‘PN40024’. This low conservation underscores the unique, species-specific transcriptomic response that has evolved in ‘GREM4’, potentially to prime systemic tissues and confer insect herbivory resistance.

#### 3.2.4 Top 20 DEGs

To elucidate temporal gene expression dynamics in systemic leaves upon insect herbivory, we compared the top 20 DEGs, ranked by |log2FoldChange|, at each timepoint between the two species (**Supplementary Table 6**). In ‘GREM4’ at 30min, 18 of the top 20 DEGs were down-regulated, including genes involved in cell wall modification (*VlTPRP-F1-1*, *VlCESA4*, *VlPE*, and *VlPRX25-like*), pathogen defense (*VlCESA4*, *VlSBT4.14/XPS1*, *VlGLIP2-1*, and *VlPRX25-like*), development (*VlnsLTP*, *VlTPRP-F1-1*, *VlStigma-specific STIG1-like protein 1*, *VlPE*, *VlSTIG1-like 1*, and *VlCucumisin*), and abiotic and wounding stress (*VlPRX25-like*, *VlKCS5*, *VlMYC70*, *VlSBT4.14/XPS1*, and *VlRD22*). Some of these genes may function as inhibitors, thus, down-regulation may enable defense pathway activation, as observed with *MYC70*, a negative regulator of cold tolerance (Ohta et al., 2018), and *CESA4*, which was more down-regulated in *Arabidopsis* resistant to *Botrytis cinerea* than the susceptible (Ramírez et al., 2011). In ‘PN40024’, 17 of the top 20 DEGs in ‘PN40024’ were up-regulated, with several linked to development (*VvEXO-1*, *VvEXO-like 2*, *VvEXO-2*, and *VvTIFY5A*), insect herbivory defense (*VvTIFY5A/JAZ8*, *VvDREB1E-like*, and *VvJAZ8*), JA (*VvTIFY5A/JAZ8*, *VvJAZ8*, and *VvTIFY5A*), brassinosteroids (*VvEXO-1*, *VvEXO-like 2*, and *VvEXO-2*), pathogen defense (*VvPUB23*, *VvERF017-2*, and *VvERF27*), generalized defense (*VvTIFY5A/JAZ8*, *VvCRK25/26*, and *VvJAZ8*), and wounding response (*VvTIFY5A/JAZ8* and *VvJAZ8*). Three up-regulated *EXO*-related genes were identified in the top 20 DEGs of ‘PN40024’ suggesting an important role in early insect herbivory response.

By 1h, in ‘GREM4’, 19 of the top 20 DEGs were up-regulated, including six linked to the insecticidal secondary metabolites flavonoids (*VlCHS/STS2*, *VlBHLH92*, *VlAMAT3*, and *VlCSH/STS5-like*), stilbenoids (*VlCHS/STS2* and *VlCSH/STS5-like*), and glucosinolates (*VlERF9-like* and *VlERF27-like*). DEGs related to JA (*VlTIFY5A/JAZ8* and *VlCYP94C1*) and SA (*VlSAMT2*) were also observed, suggesting an eminent phytohormonal influence at 1h. Notably, upon herbivory, *ERF* gene family members are known to increase in expression in resistant compared to susceptible plants (Hoseinzadeh et al., 2020; Bihani et al., 2024) while up-regulation of *TIFY5A/JAZ8* enhances insect herbivory resistance (Hoo et al., 2008; Yan et al., 2018; Li et al., 2023b). Their up-regulation, coinciding with secondary metabolite biosynthesis genes, suggests they may contribute to defense by promotion of insecticidal secondary metabolite biogenesis. In ‘PN40024’, the top 20 DEGs included JA/MeJA/JA-Ile (*VvTIFY5A/JAZ8*, *VvCYP94C1*, *VvTIFY5A*, and *VvOPR11-1*), SA/MeSA (*VvNAMT1/SAMT*), systemic acquired response (SAR) (*VvGLIP2-2*), abiotic stress response (*VvPUB23, VvOPR11-1*, *VvAVT1I-2*, *VvHSPCII* (*HSP21*), and *VvUGT85A5*), and development or cellular transport (*VvEXO-like 2*, *VvTIFY5A*, *VvCTPS1*, *VvSMR4*, *VvDTX49*, and *VvSRG1-1*) related genes.

At 4h, in ‘GREM4’, 13 of the top 20 DEGs were up-regulated. Up-regulated DEGs were associated with flavonoid biosynthesis (*VlCHS/STS4-like*), pathogen defense (*VlSPCL-like 45* and *VlEIX2*), biotic stress signaling (*VlGLUA3-like 1* and *VlCYP71E7*), SA (*VlSAMT2*), JA (*VlCYP71E7*), ETH (*VlABR1-like*) and wax and cuticle-related implications (*VlDET2/SDH*), amongst others which are related to insect defense. Down-regulated DEGs were primarily associated with pathogen defense (*VlPR10.7*), lignin and cell wall modification (*VlPRX25-like*, *VlGH9B8*, and *VlIRX12*), and seed development/nutrient storage (*VlLBP65* and *VlCYP707A2*). Despite a continued, robust transcriptomic response in ‘GREM4’ at 4h (269 DEGs), only eleven DEGs were identified in ‘PN40024’ which were mainly involved in development (*VvENOD2-2*, *VvENOD2-1*, and *VvGLUA3-like 2*) and signaling (*VvPdLRRK1* and *VvT6H22.26*). This low number of DEGs by 4h in ‘PN40024’ supports the theory that continued expression of defense signaling and secondary metabolite genes at 4h, as observed in ‘GREM4’, can positively impact herbivory defense.

The top 20 DEGs in ‘GREM4’ at 30min were mainly related to signaling and wound repair, whereas at later timepoints, the top 20 DEGs were involved in secondary metabolite biosynthesis and signaling and wound repair to a lesser degree. This differed from ‘PN40024’ top 20 DEGs, which were mostly involved in signaling and wound repair across the three time periods. Only 1 of the top 20 DEGs were involved in secondary metabolite production at 1h and 4h. This comparison of the highest expressed genes across the three timepoints highlights a major difference in the systemic responses of the two accessions; ‘GREM4’ highly expressed genes related to insecticidal secondary metabolites following 1h of herbivory, which may contribute to deterring herbivory in those tissues.

#### 3.2.5 Enrichment analyses

**Figure 5:**
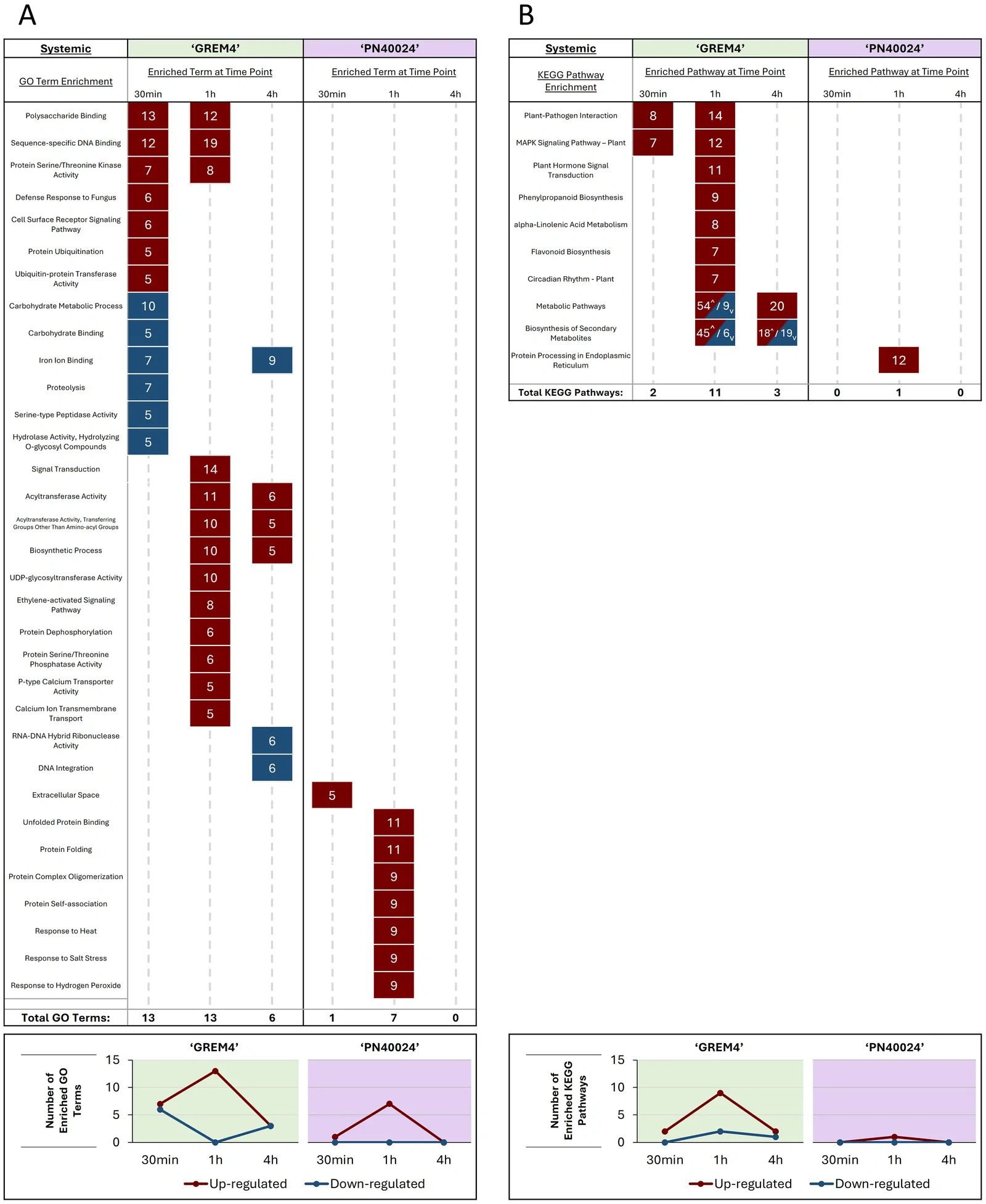
Enrichments identified of systemic DEGs in ‘GREM4’ and ‘PN40024’ at the timepoints 30min, 1h, and 4h. A box to the right of the enrichment indicates it was significantly enriched at the corresponding timepoint and in the corresponding species, both listed at the top of the column for both the GO Term **(A)** and KEGG **(B)** graphics. If a box is not present for a given enrichment at a given timepoint, the enrichment was not significantly enriched for that function or pathway at that timepoint. The color of the box signifies if the DEGs enriched for the function or pathway were up- (maroon) or down-regulated (navy); The number within the box indicates the number of DEGs implicated in the biological function or pathway. If a box is split in two, half maroon and half navy, then the enrichment was identified at that timepoint of both up- and down-regulated sets of DEGs; The leftmost number in such boxes is the number of DEGs implicated in the enrichment derived of the up-regulated DEGs while the rightmost number is the number of DEGs implicated in the enrichment derived of the down-regulated DEGs. At the base of both graphics, two line charts graphically illustrate the numbers of enrichments identified in each species, ‘GREM4’ and ‘PN40024’, over time. The red lines represent the number of enrichments of up-regulated DEGs and blue lines represent the number of enrichments of down-regulated DEGs. Significant enrichments for both GO Terms and KEGG were defined as p.adj ≤ 0.05 and ≥ 5 DEGs.

Enrichment analyses were conducted on DEGs identified in systemic response leaves and enrichments were identified. In ‘GREM4’, up- and down-regulated DEGs ranged from 131 to 419 and 41 to 138, respectively, while in ‘PN40024’ up- and down-regulated DEGs ranged from eight to 64 and two to three DEGs, respectively (**Table 1**; **Figure 4A**). Enrichment analyses of these gene sets revealed up-regulated DEGs in ‘GREM4’ systemic leaves were involved in plant-pathogen interaction and signaling pathways at 30min, as seen by functional enrichments of DEGs involved in DNA binding, ubiquitination, and protein kinase activity (**Figure 5**, **Supplementary Tables 4**, **5**). Down-regulated genes at 30min were enriched for carbohydrate metabolic processes, iron ion binding, proteolysis, hydrolase activity, and calcium transport, which preserve resources under stress. Up-regulated DEGs detected at 1h were further enriched for genes involved in hormone signaling and secondary metabolite biosynthesis pathways, including genes functioning in signal transduction, acetyltransferase activity, UDP-glycosyltransferase activity, phenylpropanoid biosynthesis, and flavonoid biosynthesis, with DEGs involved metabolic pathways and secondary metabolite biosynthesis persisting at 4h. A clear pattern of up-regulated DEGs involved signaling within the first 30min-1h, followed by DEGs involved in secondary metabolite biosynthesis at 1h and 4h was observed in ‘GREM4’ in systemic leaves. ‘PN40024’ DEGs did not have significant pathway enrichments at 30min or 4h, but up-regulated DEGs involved in protein processing were enriched at 1h, including genes involved in protein folding and response to abiotic stress. The general lack of enrichments identified in ‘PN40024’ were likely due to the lack of DEGs identified in systemic leaves. Overall, ‘GREM4’ had more DEGs at each timepoint compared to ‘PN40024’, which were enriched for signaling and secondary metabolite biosynthesis functions, likely priming systemic tissues to enhance insect herbivory defense.

In conclusion, ‘GREM4’ induced transcription of defense-related DEGs, especially in secondary metabolite biosynthesis, signaling, and plant-pathogen interactions, across herbivory-specific, systemic-specific, and conserved gene sets, indicating a robust defense response. In ‘PN40024’, secondary metabolite and signaling related pathways were also enriched, but were more restricted to herbivory leaves and there were fewer DEGs than was observed in ‘GREM4’, suggesting a weaker systemic defense.

### 3.3 Herbivory compared to systemic response

To reveal tissue-specific differences in response to insect herbivory between herbivory and systemic leaves, we compared the transcriptomes between these two tissues in ‘GREM4’ and ‘PN40024’.

#### 3.3.1 Herbivory and systemic results comparisons

First, we compared the results from herbivory compared to control and systemic compared to control comparisons to one another.

##### 3.3.1.1 DEGs and DEG conservation

**Figure 6:**
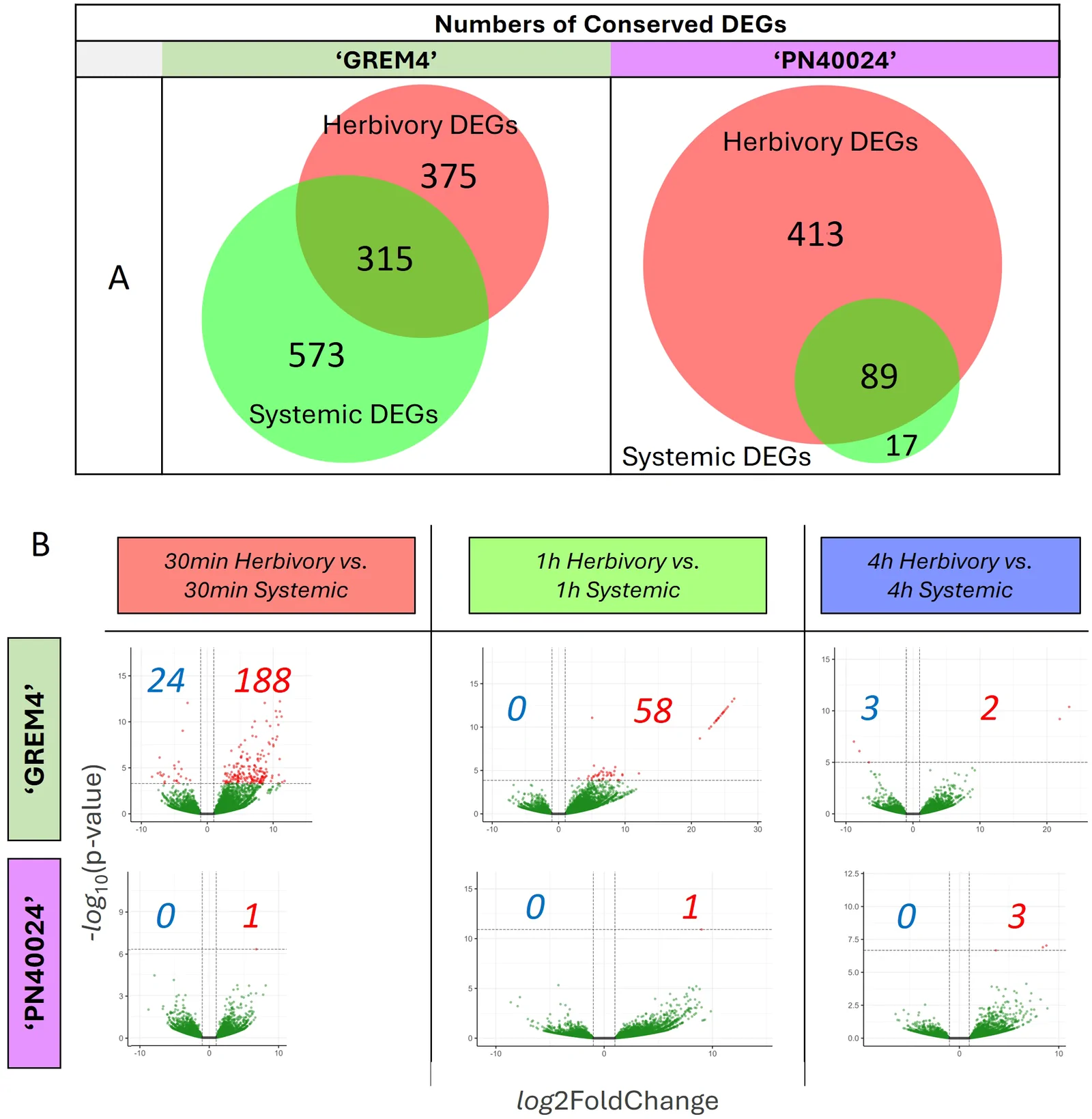
Gene expression results from herbivory compared to systemic leaf comparisons after 30min, 1h, and 4h of Japanese beetle herbivory. **(A)** Venn diagrams in ‘GREM4’ and ‘PN40024’ depicting the number of overlapping and unique DEGs between herbivory and systemic leaves when all up- and down-regulated DEGs across all time points were combined. **(B)** Volcano plots depicting numbers of genes with significantly different expression between herbivory and systemic leaves in ‘GREM4’ and ‘PN40024’ at time points of 30min, 1h, and 4h. DEGs were defined using an |log2FoldChange| ≥ 2 and a p.adj value ≤ 0.05 to determine significance; Grey dashed lines demarcate these thresholds in the plots. The y-axis plots p-values for each datapoint for illustrative purposes. Red dots represent genes with an |log2FoldChange| value ≥ 2 and a p.adj value of ≤ 0.05. Green dots represent genes with an |log2FoldChange| value ≥ 2 and a p.adj value of > 0.05. Grey dots represent genes with an |log2FoldChange| value < 2 and a p.adj value of > 0.05. Red and blue inlaid numbers report number of DEGs with greater, or lower, expression in herbivory compared to systemic leaves, respectively.

In ‘GREM4’, when all timepoints and up- and down-regulated DEGs were combined, a greater number of total non-redundant DEGs were identified in systemic (888) compared to herbivory (690) leaves (**Figure 6A**) which was surprising. Systemic leaves exhibited a higher proportion of down-regulated genes at each timepoint. In ‘PN40024’, the vast majority of DEGs were up-regulated. Fewer DEGs were identified in systemic (106 non-redundant DEGs) compared to herbivory leaves (502), reporting a weaker response to insect herbivory in systemic leaves of ‘PN40024’. Importantly, ‘PN40024’ exhibited fewer total DEGs, in both herbivory and systemic leaves, compared to ‘GREM4’. In summary, the numbers of DEGs in herbivory and systemic responses were notably higher in ‘GREM4’, suggesting a more robust transcriptomic response.

In ‘GREM4’, when all timepoints and up- and down-regulated DEGs were combined, 315 of 1,263 total non-redundant DEGs (25%) were conserved across both herbivory and systemic responses (**Figure 6A**; **Supplementary Figure 3A**), whereas 573 were identified as systemic-specific and 375 were herbivory-specific DEGs. In ‘PN40024’, 17% (89 of 519) of total non-redundant DEGs were conserved between both responses (**Figure 6A**; **Supplementary Figure 3B**), while 17 were systemic-specific and 413 were herbivory-specific DEGs. These results suggest that the systemic response of ‘PN40024’ by-in-large overlaps the herbivory response upon insect herbivory response, but the herbivory response itself is more complex. ‘GREM4’ had 573 DEGs that were systemic-specific, suggesting that the systemic response is more distinct from the herbivory response in ‘GREM4’ compared to ‘PN40024’.

#### 3.3.2 Direct transcriptomic comparisons

Next, transcriptomes of herbivory and systemic tissues were directly compared using DESeq2 to assess absolute expression differences between responses.

##### 3.3.2.1 DEG identification

In ‘GREM4’, 188 genes were more highly expressed in herbivory than systemic leaves after 30min, while 24 exhibited lower expression (**Figure 6B**); By 1h, these numbers declined to 58 and 0, and by 4h to only 2 and 3, respectively. These results indicate that initial large transcriptomic differences between herbivory and systemic tissues subside over time to the point where they are not significantly different by 4h. In contrast in ‘PN40024’, only 5 DEGs were detected across all timepoints, suggesting little to no transcriptomic distinction between herbivory and systemic tissues.

## 4 Discussion

Defenses have evolved in plants to mitigate the adverse effects of biotic stressors, including insect herbivory. This study investigated the direct and systemic transcriptomic responses of insect-resistant ‘GREM4’ and insect-susceptible ‘PN40024’ grapevine leaves to herbivory at 30min, 1h, and 4h. The objective was to elucidate temporal, spatial, and species-specific dynamics of early (≤4h) plant responses to insect herbivory.

### 4.1 Numbers of DEGs in herbivory leaves increase over time in ‘GREM4’ but decrease in ‘PN40024’

Though previous studies have made excellent strides in defining the responses of plants to insect herbivory, many of these studies focused on gene expression within timeframes spanning greater than one day, leaving the early responses of resistant and susceptible plants under-examined (Broekgaarden et al., 2007; Sarosh and Meijer, 2007; Pandey et al., 2008; Ehlting et al., 2008; Coppola et al., 2013; Appel et al., 2014; Wang et al., 2014; Tzin et al., 2015, Tzin et al., 2017; Huang et al., 2015; Li et al., 2016, Li et al., 2023b; Liu et al., 2016; Tetreault et al., 2019; Chen et al., 2019, Chen et al., 2022; Zhang et al., 2020; Dixit et al., 2020; Gyan et al., 2020; Malhotra et al., 2022; Xue et al., 2022; Zhuang et al., 2022; Jan et al., 2022; Jing et al., 2024). This considerable knowledge gap is addressed in our study by comparing the transcriptomic responses of resistant ‘GREM4’ and susceptible ‘PN40024’ under Japanese beetle herbivory at 30min, 1h, and 4h.

Resistant plants typically exhibit more DEGs in response to insect herbivory than susceptible plants. Signal transduction and defense genes are frequently differentially regulated upon biotic stress in aptly responsive plants (Mostafa et al., 2022). In ‘GREM4’, nearly twice as many DEGs were detected at 1h and three times as many at 4h compared to ‘PN40024’. Similar patterns have been observed in other species, including cotton, where resistant lines exhibited >1,000 and >200 DEGs than susceptible lines after 24h and 48h of whitefly (*Bemisia tabaci*) herbivory (Li et al., 2016). In cassava (*Manihot esculenta*) whiteflies induced 740 uniquely up- and 730 down-regulated DEGs in the resistant compared to susceptible line after 1d of herbivory (Nye et al., 2023).

In ‘GREM4’, the number of DEGs increased over time peaking at 4h in herbivory leaves, whereas, in ‘PN40024’, the number of DEGs peaked at 30min and decreased thereafter. Similar trends in resistant plants have been observed at longer time scales, with DEGs peaking beyond 4h. For example, in resistant cotton, peak numbers of DEGs occurred at 48h post-herbivory upon both cotton bollworm (*Helicoverpa armigera*) (Huang et al., 2015) and whitefly (Li et al., 2016) herbivory. The number of DEGs in response to insect herbivory in ‘GREM4’ may continue to increase beyond the 4h timepoint, but this will need to be further tested.

### 4.2 Greater transcriptomic response to insect herbivory observed in ‘GREM4’ systemic leaves compared to ‘PN40024’

Systemic response is likewise crucial for plant defense. In this study, 1,120 DEGs were identified in systemic leaves of ‘GREM4’ following Japanese beetle herbivory, compared to only 116 in ‘PN40024’. Previous studies have reported large numbers of DEGs in non-afflicted leaves during insect herbivory, such as in tea infested by tea geometrid, where 558 systemic DEGs were identified across timepoints (3–24h) (Zhou et al., 2020), and in *Arabidopsis*, where insect herbivory induced hundreds to thousands of systemic DEGs at 6h or 24h, depending on the insect species (Appel et al., 2014; Xue et al., 2022). Crucially, resistant plants often exhibit stronger systemic transcriptomic responses than susceptible counterparts, as seen in maize, where 5,070 systemic DEGs were identified in resistant (primed) leaves versus 3,689 in susceptible (unprimed) (ul Malook et al., 2021). Few studies have compared systemic DEGs between resistant and susceptible species, especially over time. Our results highlight that resistant ‘GREM4’ exhibited a stronger systemic transcriptomic response, which is perpetuated over time (0-4h), compared to susceptible ‘PN40024’. It is likely that systemic signaling plays an integral role in this resistance, conferring defense signals to distal tissues, resulting in enhanced expression of defensive secondary metabolite genes throughout, improving defense.

In systemic responses that enhance insect herbivory resistance, activation of key pathways induce accumulation of metabolites which heighten defense, a phenomenon known as ‘priming’ (Schilmiller and Howe, 2005). For instance, Northern armyworm (*Mythimna separata*) that fed on primed maize leaves had >50% lower mass compared to those that fed on unprimed leaves; the decrease was also inversely correlated with BXD and JA accumulation (ul Malook et al., 2021). Similarly, rice leafrollers (*Cnaphalocrocis medinalis*) that fed on systemic rice tillers of primed versus unprimed plants for 2d had ~50% reduced mass, also linked to elevated JA levels (Tong et al., 2023). Defense signaling phytohormone JA appears to be a critical component in priming, as is the systemic accumulation of insect defense-related metabolites including terpenes, phenylpropanoids, and trypsin protease inhibitors as observed in cotton (Mamin et al., 2023), soybean (Dryburgh and Davis, 2021), husk tomato (Meza-Canales et al., 2022), maize (ul Malook et al., 2019), and *Arabidopsis* (Xue et al., 2022).

Species with strong constitutive defenses often exhibit milder inducible transcriptomic responses to stress, an evolutionary adaptation conserving finite energetic resources by avoiding superfluous defense activation (Rasmann et al., 2015; Whitehill et al., 2021; Zhang et al., 2022b; Lv et al., 2023). However, a previous study reported that ‘GREM4’, not ‘PN40024’, exhibits stronger constitutive defenses (Dixon and Gschwend, 2024). ‘PN40024’ had fewer DEGs in response to herbivory, compared to ‘GREM4’ in general, which decreased over time, likely hampering defense.

Resistance-related compounds often compromise grape quality and taste (Chambers et al., 2013; Black et al., 2014; Zheng et al., 2022). Considering grape flavor, quality, sugar content, and other characteristics are particularly crucial to wine production, we hypothesize that artificial selection for favorable grape flavor profiles in *V. vinifera*, followed by successive vegetative (clonal) propagation may have inadvertently curtailed resistance. Grapevine domestication began 6,000-8,000 years ago, with selection for high yield, rapid growth, berry size, sugar content, and hermaphroditic flowers, but also resulted in the loss of resistance traits (Grassi and Arroyo-Garcia, 2020; Chaudhary, 2013). To maintain the genetic composition contributing to desirable traits within a cultivar, cultivated grapevine is vegetatively propagated. As a result, the majority (75%) of the *V. vinifera* cultivars in the USDA grape germplasm collection are related by a first-degree relationship to at least one other cultivar (Myles et al., 2011). Vegetative propagation has put these elite cultivars at a disadvantage in the evolutionary ‘arms race’ between plants and insect herbivores, reducing opportunities for the introduction of new genetic diversity that contributes to novel defensive mechanisms. The use of wild grapevines, which have co-evolved with present insect pests, can help to overcome this challenge. For example, the introduction of *Phylloxera* to Europe in the mid-20^th^ century, invoked a strong selective pressure on highly susceptible *V. vinifera* across the continent, until inter-specific rootstocks were introduced (This et al., 2006). Wild grapevine genetics have also been utilized to breed downy and powdery mildew resistant grapevine cultivars (Merdinoglu et al., 2018). Our study supports the use of wild grapevines as a genetic source for breeding and crop improvement efforts to enhance herbivory resistance in grapevine.

### 4.3 Expression of key signaling and secondary metabolite biosynthesis pathway genes contribute to ‘GREM4’ defense response

When reviewing differences in the DEGs between treatments and species, we identified clear trends. Over time, signaling-related DEGs preceded the activation of secondary metabolite genes in systemic leaves in ‘GREM4’. At 30min, many of the top 20 up-regulated DEGs in ‘GREM4’ herbivory leaves were involved in signaling, but at 30min in systemic tissues, many of the top 20 DEGs were down-regulated and involved in cell wall biosynthesis and development. Meanwhile, at 1h and 4h, DEGs in both systemic and herbivory leaves were enriched for signaling and secondary metabolite biosynthesis, including genes involved in phytohormones, and MAPK signaling, flavonoid, terpene, and phenylpropanoid biosynthesis. Of the top 20 DEGs in ‘GREM4’ herbivory leaves at 30min, 1h, and 4h, 27% were found to also be a top 20 DEG in 30min, 1h, or 4h in systemic leaves. In ‘PN40024’, over half of the top 20 DEGs in both herbivory and systemic leaves were involved in signaling and phytohormones at 30min while at 1h the majority in both herbivory and systemic leaves were related to signaling, cell wall biogenesis, growth, and response to abiotic stress. By 4h, herbivory leaves had a high proportion of genes involved in signaling, secondary metabolism, and carbon metabolism, but systemic DEGs did not have significant functional enrichment. These results suggest that high expression of secondary metabolite-related genes play an important role in conferring resistance.

Interestingly, some of the top 20 DEGs of the herbivory and/or systemic leaves had one or more homologs also in the top 20 DEGs. For example, ‘GREM4’ herbivory leaves had three cytochrome P450 genes (*VlCYP71E7*, *VlCYP716A1*, *VlCYP-like*), two GDSL lipase enzyme genes (*VlGLIP1* and *VlGLIP2-1*), two glycine-rich protein paralogs (*VlGRP5-like* and *VlGRP5-like 2*), and five stilbene biosynthesis paralogs (*VlSTS1-3*, *VlSTS1-4*, *VlSTS1-5*, *VlSTS1-6*, and *VlSTS1-7*). The high expression of five stilbene biosynthesis genes in herbivory tissues suggest these genes may play a pivotal role in deterring insect herbivory, as stilbenes can be toxic to insects (Gabaston et al., 2018). The top 20 DEGs of systemic leaves in ‘GREM4’ also exhibited homologs, including *VlBHLH92* and *VlBHLH167* transcription factors, three flavonoids/stilbene biosynthesis genes (*VlCHS/STS2*, *VlCHS/STS4-like*, and *VlCHS/STS5-like*), three cytochrome p450 genes (*VlCYP71E7*, *VlCYP707A2*, and *VlCYP94C1*), two ethylene response factors (*VlERF9-like* and *VlERF27-like*), two *STIG1-like 1* paralogs, and two *TPRP-F1* paralogs. The systemic leaves also expressed genes related to stilbene and flavonoid production, but these genes were different than those expressed in the herbivory tissues. These results demonstrate that gene homologs can have specialized expression that contributes to a robust herbivory response.

Our study revealed stark contrasts in functional enrichments between resistant ‘GREM4’ and susceptible ‘PN40024’ systemic DEGs. Of ‘GREM4’ DEGs, numerous functional and pathway enrichments impacting resistance and response to herbivory damage were observed, including signaling (calcium ion signaling, lipid transport, and MAPK signaling), phytohormones, acyltransferase-related, and oxidative stress regulation terms. DEGs related to these terms likely contribute broadly to stress responses beyond pathogen defense, post-translational modifications (such as those involving acyltransferase-related DEGs) enhancing defense activity, and regulation of oxidative stress indirectly strengthening plant defense. Notably, ‘GREM4’ systemic DEGs were enriched for several pathways, including ‘flavonoid biosynthesis’, ‘phenylpropanoid biosynthesis’, pathogen response, and signaling (including MAPK signaling and phytohormones). These pathways are linked to insect defense and have been observed in systemic herbivory responses in wild pigeonpea (Malhotra et al., 2022), *Arabidopsis* (Appel et al., 2014; Toyota et al., 2018; Xue et al., 2022), tea (Zhou et al., 2020), and others (Schilmiller and Howe, 2005), including resistant species (Malhotra et al., 2022). In contrast, enrichments of ‘PN40024’ DEGs were limited to protein remodeling. These findings highlight the robust, and diverse, systemic signaling and defensive capacity of ‘GREM4’, in contrast to the weaker response of ‘PN40024’.

### 4.4 Pathways and genes with dichotomous expression between ‘GREM4’ and ‘PN40024’

#### 4.4.1 Terpenes

‘GREM4’ herbivory DEGs were enriched for terpene-related pathway at 1h and 4h, but were not an enriched at any timepoint for ‘PN40024’. Of specific interest, *VlTPS14–1* was the 7^th^, 40^th^, and 5^th^-most up-regulated DEG at 30min, 1h, and 4h, respectively, in ‘GREM4’, while its ortholog in ‘PN40024’, *VvTPS14-1*, was not a DEG at any timepoint. *TPS* genes are critical to many steps of terpene biosynthesis (Sun et al., 2022; Li et al., 2023a) and are important for insect defense, as silencing *TPS* in rice increased susceptibility to bird cherry-oat aphid while over-expressing enhanced resistance (Sun et al., 2017). Further, *TPS* genes are known to be insect herbivory inducible as eight *GhTPS* genes were up-regulated in cotton upon concurrent cotton bollworm and small green plant aphid (*Apolygus lucorum*) herbivory (Huang et al., 2018), meanwhile, tobacco (*Nicotiana benthamiana*) over-expressing *GhTPS12* demonstrated increased resistance to both insects compared to WT (Huang et al., 2018). These results suggest that terpene biosynthesis, and specifically *VlTPS14-1*, plays an important role in ‘GREM4’ resistance.

#### 4.4.2 JA-related genes

*JAZ8* is crucial in JA biosynthesis during wounding (Hoo et al., 2008; Zhang et al., 2019) and insect herbivory (Hoo et al., 2008; Yan et al., 2018). In *Arabidopsis*, Ca^2+^ signaling, triggered by wounding from beet armyworm herbivory, emanates from the site of damage resulting in phosphorylation of JAV1 in the JAV1-JAZ8-WRKY51 (JJW) complex, inactivating the JJW complex (a transcriptional inhibitor of JA genes) and promoting JA-related gene expression within 1h in both local and systemic tissues (Yan et al., 2018). JAZ8, while it cannot bind to DNA directly (Thines et al., 2007), is a repressive co-factor in the JJW, enhancing its binding affinity to the promotor of *AOS* (a critical JA biosynthesis gene), thereby repressing *AOS* expression and JA biosynthesis (Kongrit et al., 2007; Yan et al., 2018). In our study, *VlJAZ8* was only detected as a DEG in ‘GREM4’ herbivory leaves at 4h (85^th^ ranked) and *VlAOS1* was up-regulated in ‘GREM4’ herbivory leaves at all timepoints, increasing from 30min to 4h, and was also a DEG in systemic leaves at 1h and 4h. Furthermore, *VlAOS3* exhibited an exceptionally high log2FoldChange (24.39) at 1h in ‘GREM4’ herbivory leaves. On the other hand, *VvJAZ8* in ‘PN40024’ was an up-regulated DEG at 30min in both herbivory (12^th^) and systemic (11^th^) leaves, and while *VvAOS1* was detected as differentially expressed at 30min and 4h in herbivory leaves, and at 1h in systemic, *VvAOS3* was not a DEG in ‘PN40024’. These results could suggest early expression of *VvJAZ8* in ‘PN40024’ at 30min may inhibit JA biosynthesis, impairing defense mechanisms and systemic signal transduction – a possible consequence of the lack of JA (Tong et al., 2023). The regulation of *VlJAZ8*, *VlAOS1*, and *VlAOS3* may be pivotal in the herbivory and systemic responses of resistant ‘GREM4’, although this hypothesis requires further validation.

#### 4.4.3 Calcium signaling

*Glutamate-receptor* (*GLR*) gene family, known for increasing calcium ion concentrations to facilitate defense signals (Toyota et al., 2018) is crucial for resistance, JA production, MAPK signaling, and phenylpropanoid biosynthesis (Xue et al., 2022). In ‘GREM4’, four of 14 *GLR2* genes — *VlGLR2*, *VlGLR2.1*, *VlGLR2.7*, and *VlGLR2.9-like* — were uniquely up-regulated in systemic leaves within 30min to 1h of herbivory. ‘GREM4’ systemic DEGs were also exclusively enriched for calcium ion signaling. This highlights the significance of differential expression of signaling-related genes in ‘GREM4’ compared to ‘PN40024’.

#### 4.4.4 Flavonoids

Flavonoids are widely recognized for their insect repellent and insecticidal properties (Kariyat et al., 2019; Chatterjee et al., 2022; Chen et al., 2022; Zhang et al., 2022a). For instance, transgenic sorghum (*Sorghum bicolor*) which produced elevated levels of flavonoids resulted in increased fall armyworm (*Spodoptera frugiperda*) mortality, compared to WT, with flavonoid extracts found to be insecticidal (Chatterjee et al., 2022). Additionally, upon brown planthopper (*Nilaparvata lugens*) feeding, resistant rice cultivars produced significantly greater quantities of flavonoids than susceptible (Zhang et al., 2022a). In our study, flavonoid-related DEGs were up-regulated in herbivory leaves of both ‘GREM4’ and ‘PN40024’, but temporal differences were identified. ‘PN40024’ exhibited up-regulation of flavonoid-related DEGs predominantly at 30min, and diminished thereafter, while ‘GREM4’ exhibited sustained expression from 1h onwards, likely contributing to enhanced herbivory resistance. Additionally, flavonoid-related pathways in systemic leaves were solely enriched of ‘GREM4’ DEGs, highlighting a species-specific flavonoid systemic response.

Key flavonoid biosynthesis genes, including *VlFLS/F3H* and *VlFLS2*, were DEGs across all timepoints in herbivory and systemic leaves of ‘GREM4’, and have been previously linked to herbivory resistance. Overexpression of *OsF3H* in rice, for instance, increased flavonoid accumulation and reduced whitebacked planthopper (*Sogatella furcifera*) herbivory (Jan et al., 2020), while *FLS* up-regulation was associated with resistance in wild pigeonpea against cotton bollworm (Malhotra et al., 2022) and in cassava upon two-spotted spider mite (TSSM) (*Tetranychus urticae*) herbivory (Chen et al., 2022). In hybrid grapevine (*V. vinifera* × *V. labrusca*), flavonoid biosynthesis genes, including *Kyoho_F3M* and *Kyoho_FLS*, were up-regulated after 72h of oriental longheaded grasshoppers (*Acrida chinensis*) feeding, in addition to increased flavonoid accumulation (Jia et al., 2022).

In our study, several flavonoid biosynthesis genes, particularly *CHS/STS* genes, exhibited timepoint-specific expression, highlighting their unique role in insect herbivory defense (Dixit et al., 2020; Tyagi et al., 2022; Jing et al., 2024). In ‘GREM4’, *VlCHS/STS2* was a DEG solely in 1h herbivory and systemic leaves while *VlCHS/STS4-like* was a DEG solely in 1h herbivory and 4h systemic leaves, suggesting discreet temporal control over flavonoid biosynthesis in response to stress. Other paralogous genes, like *VlCHS/STS5-like*, exhibited broader expression across timepoints as it was a DEG at 1h and 4h in herbivory and at 30min and 1h in systemic. These results indicate the expansion of gene families, paired with subsequent evolution/diversification of novel members, give rise to unique regulatory and transcriptomic profiles with differential temporal-, spatial-, and stress-specific expression between species, which, in the case of ‘GREM4’, likely positively impacts resistance.

#### 4.4.5 Stilbenes

Stilbenes, non-flavonoid polyphenols (Gao et al., 2024), have in some cases been found to enhance biotic resistance (Eisenmann et al., 2019). In 2008 *Stilbene synthase* (*STS*) was successfully cloned from a wild *Vitis* species and transferred to *V. vinifera* cv. ‘Thompson Seedless’ to imbue fungal resistance (Fan et al., 2008). While some positive impacts of stilbenes on herbivory resistance in *Vitis* have been reported against mites (Javadi Khederi et al., 2018) and *Phylloxera* (Eitle et al., 2019), deeper investigation is still required. In our study, five *STS-*gene family members were identified in the top 20 DEGs of ‘GREM4’ after 4h of insect herbivory. This suggests a role in insect defense. Interestingly, all five noted *VlSTS1* genes were paralogous, thus, these genes may hypothetically have been retained in the ‘GREM4’ genome evolutionarily due to their role in stress-specific response to insect herbivory. Further, we reported that ‘GREM4’ herbivory DEGs were enriched for stilbene pathways, but not ‘PN40024’. Previous studies in fall armyworm *in vitro* (McComic et al., 2021) and *Gonipterus platensis* in *Eucalyptus* (Campos et al., 2022) report the insecticidal nature of stilbenes, but toxicity can vary. Our study supports the premise that stilbenes enhance insect resistance *in planta*, especially considering their decreased presence as DEGs in ‘PN40024’.

#### 4.4.6 Phenylpropanoids

The phenylpropanoid pathway is a critical upstream pathway of many secondary metabolites critical to plant defense including SA, stilbenes, coumarins, lignins, and flavonoids (La Camera et al., 2004; Chang et al., 2019). The first three steps of the pathway are catalyzed by the enzymes PAL, C4H, and 4CL, respectively (La Camera et al., 2004). In our study, *C4H* was identified as an up-regulated DEG in ‘GREM4’ 4h herbivory samples, speaking to its importance.

Phenylpropanoid-related genes, particularly *PAL*, are regularly up-regulated in response to insect herbivory. In rice, significant up-regulation of phenylpropanoid pathway genes, including *OsPAL6*, *OsPAL7*, *Os4CL6*, and *OsSHT1*, were identified when comparing 3h to 30min of rice leafroller herbivory (Zhuang et al., 2022). Meanwhile, *PAL* expression in cotton persists even after 4d of feeding (Dixit et al., 2020) while a similar response is observed in hybrid grapevine after 72h of oriental longheaded grasshopper herbivory (Jia et al., 2022). Additionally, in some species, expression of phenylpropanoid-related genes is positively correlated with herbivory resistance, such as observed in a screen of 29 carrot (*Daucus carota*) lines with *DcPAL1* and *DcPAL3* expression (Simlat et al., 2013) or in cassava where DEGs of resistant lines exhibited enrichment for phenylpropanoid and flavonoid biosynthesis, along with increased accumulation of such metabolites, upon TSSM herbivory compared to susceptible lines (Chen et al., 2022). These findings reinforce the role of *PAL1* in insect resistance.

Dixon and Gschwend (2024) found the *PAL1* gene family was larger in ‘GREM4’ (twelve members) compared to ‘PN40024’ (four), with five of the eight additional family members significantly up-regulated upon insect herbivory in ‘GREM4. In our current study, *PAL1* gene family members in ‘GREM4’ again exhibited species- and timepoint-specific expression in systemic leaves (**Supplementary Figure 4**). *VlPAL1–4* was an up-regulated DEG at all timepoints, *VlPAL1–8* at 30min and 1h, and *VlPAL1–1* and *VlPAL1–7* at 1h. Meanwhile, in ‘PN40024’, *VvPAL1* genes were not differentially expressed. Considering *VlPAL1-4*, *VlPAL1-7*, and *VlPAL1–8* are paralogs in ‘GREM4’, *PAL1* presents another case study in ‘GREM4’ of gene family expansion and divergence likely enhancing resistance. Further, phenylpropanoid-related enrichments were observed of herbivory DEGs at 1h and 4h, systemic DEGs at 1h, and were enriched of herbivory-specific, systemic-specific, and conserved-in-both gene sets from the overlap analysis, exemplifying the ubiquitous nature of phenylpropanoids in the ‘GREM4’ response. In stark contrast, phenylpropanoids were not enriched in ‘PN40024’ DEG genesets.

Increased expression of genes in the phenylpropanoid pathway, particularly *VlPAL*, likely plays an important role in supplying reactants for downstream processes vital to stout insect herbivory resistance in ‘GREM4’.

#### 4.4.7 Uncharacterized genes

It is important to mention that some of the top differentially expressed genes in ‘GREM4’ were uncharacterized and did not have an ortholog in ‘PN40024’. For example, *VITVlaGREM4_v1.0.un.ver1.0.g140.9* was ranked the 1^st^, 29^th^, and 4^th^-most and 29^th^, 37^th^, and 6^th^-most differentially expressed gene in herbivory and systemic leaves at 30min, 1h, and 4h, respectively, in ‘GREM4’. Expression data for *VITVlaGREM4_v1.0.un.ver1.0.g140.9* across all tissues and timepoints can be found in **Supplementary Table 7**. No putative function could be predicted based on homology to other known genes. As such, *VITVlaGREM4_v1.0.un.ver1.0.g140.9* may play a unique role in ‘GREM4’ defense, and thus, is an excellent candidate for future study.

### 4.5 Future directions

Our study identified many candidate genes that show promise of conferring insect herbivory resistance in ‘GREM4’, but future studies are required to validate these assertions, as transcriptomic alterations alone do not necessarily result in phenotypic or functional changes. For example, genes such as *VlTPS14-1*, *VlFLS/F3H*, *VlFLS2*, *VlPAL* and *VITVlaGREM4_v1.0.un.ver1.0.g140.9* should be functionally investigated via overexpression or knock-out studies to determine the ramifications of these target genes on the insect resistant phenotype. Additionally, downstream studies, such as broad-spectrum metabolomics, can investigate metabolite families revealing overall trends in secondary metabolites that change in abundance upon insect herbivory in resistant versus susceptible grapevine germplasm. Targeted metabolomics can be used to explore accumulation of specific metabolites, such as phenylpropanoids or specific monoterpenes, to quantify their presence in herbivory relative to systemic leaves. Spectroscopy or crystallography could explore unique secondary or tertiary structure which could result in enhanced activity of paralogous DEGs in ‘GREM4’ which are up-regulated under insect herbivory, such as *VlGLUA3-like 1*. Intriguing systemic response findings were also identified as 1,120 systemic DEGs were identified in ‘GREM4’ compared to only 116 in ‘PN40024’. While this suggests defensive priming in ‘GREM4’, follow-up insect herbivory feeding studies are necessary to verify this assertion. Together, these future studies can serve to further understand the molecular responses of grapevines under insect herbivory stress.

### 4.6 Conclusions

This study revealed distinct temporal and spatial differences in the transcriptomic responses of ‘GREM4’ and ‘PN40024’ to Japanese beetle herbivory. In ‘GREM4’, the number of DEGs increased over time in both herbivory and systemic leaves, while, in ‘PN40024’, they decreased. ‘GREM4’ herbivory leaves exhibited strong activation of genes associated with insect repellent and insecticidal activities, including terpenes, phenylpropanoids, and flavonoids, many of which did not have an ortholog in ‘PN40024’. In contrast, the herbivory response in ‘PN40024’ was characterized by genes related primarily to pathogen defense, abiotic stress, phytohormones, and growth and development. Notably, ‘GREM4’ systemic leaves demonstrated early activation of signaling-related genes, which preceded the up-regulation of insect defensive secondary metabolite pathways, including flavonoids and phenylpropanoids. This “priming” of systemic leaves in ‘GREM4’ likely contributed to enhanced resistance to insect herbivory in these tissues. In ‘PN40024’, although phytohormonal signaling and pathogen defense genes were activated in systemic leaves, there was a marked lack of genes involved in secondary metabolite biosynthesis, far fewer genes related to signaling, and a general lack of DEGs overall, resulting in few defense-related enrichments. These findings report that the enhanced insect herbivory resistance of ‘GREM4’ is likely due, in part, to a robust and temporally-dynamic transcriptomic response within the first 4 hours of insect herbivory in both fed-upon and distal tissues, particularly in secondary metabolite biosynthesis, whereas ‘PN40024’ exhibited a more subdued transcriptomic response, likely diminishing defense. Functional validation studies, metabolomic analyses, and defensive priming insect herbivory challenge assays are necessary to confirm these conclusions. These influential findings could help leverage the genetic diversity inherent to wild grapevine to improve insect resistance in cultivated *Vitis* varieties and other crops.

## Data Availability

The raw transcriptomic data used in this project can be accessed via NCBI BioProject number PRJNA1070606. The genome annotation nomenclature used for this project is available at https://www.grapegenomics.com/pages/VlaGREM4/ and https://github.com/cdixo/Vitis-labrusca-Version-2-Genome-Annotation.git. Custom codes used for data analyses are provided at https://github.com/cdixo/Inter-species-and-Herbivory-Publication.git.
