# Supplementary material for: Insect herbivory time-course reveals unique temporal transcriptomic responses in resistant and susceptible grapevines
Source: Front Plant Sci. 2026 Aug 19;17:1874271. doi: 10.3389/fpls.2026.1874271 (PMC13533947; doi:10.3389/fpls.2026.1874271)
Supplement: Supplementary file 1 [file Supplementaryfile1.docx]

**Supplementary Figures**

**Fig. S1** Insect Herbivory Leaf Images

**Fig. S1 Description –** Images from the herbivory time course study which generated the insect-herbivory-afflicted, systemic, and control leaf tissues which were sequenced for our transcriptomic study herein. A. Leftmost Picture – Placing a mesh bag over a grapevine leaf prior to placing a beetle within to start an experimental run. All leaves were mature and of the same size as they had to fit within the bag without folding. Rightmost Picture – A Japanese beetle in a bag during a feeding run. B. Representative images depicting damage from Japanese beetles in ‘GREM4’ and ‘PN40024’ leaves from the herbivory time course study. Images show feeding after 30min, 1h, and 4h. Arrows indicate locations of feeding damage.

**Fig. S2** DEG Conservation (Venn Diagrams) In Transcriptomic Responses

**Fig. S2 Description -** Venn diagrams depicting the number of overlapping and unique DEGs between groups of DEGs in various comparisons. Presented are Venn diagrams generated using up-regulated DEGs only (‘Up’), down-regulated DEGs only (‘Down’), and when all up- and down-regulated DEGs are combined (‘All’). The ‘All’ comparison venn diagram for A-D can also be found in other figures but are presented here as well for comparative purposes. Comparisons presented herein include herbivory compared to 0h (A & B), systemic compared to 0h (C & D), and herbivory compared to systemic (E & F). F. No DEGs were conserved between any time point for herbivory compared to systemic comparisons in ‘PN40024’, therefore, all DEGs were unique and no venn diagrams were created.

**Fig. S3** DEG Conservation (Upset Plot) In Transcriptomic Responses

**Fig. S3 Description -** Upset plot illustrating the intersection (or overlap) among all sets of DEGs simultaneously. For this analysis, up- and down-regulated DEGs in herbivory and systemic responses were not combined and then further broken down by timepoint. Subsequently, these lists were simultaneously queried for overlaps, the results seen above. ‘GREM4’ (A) and ‘PN40024’ (B) comparisons were conducted independently. All comparisons were compared against 0h (control). Numbers of DEGs in each comparison (size of the lists of gene names) can be seen in the bottom left horizontal bar graph. Numbers of DEGs conserved, or unique, can be seen in the vertical bar graph, center. Single, unconnected dots in the matrix represent DEGs which were exclusively identified within only the noted comparison while dots connected by lines illustrate conservation of DEGs between the connected comparisons. Red indicates the set of genes is an up-regulated DEG dataset while blue indicates the gene set is a down-regulated DEG dataset. Graphic designed via UpSetR.

**Fig. S4** *PAL1* Gene Family Expression in Systemic Leaves

**Fig. S4 Description –** *PAL1* gene family members’ expression under insect herbivory in systemic leaves of ‘GREM4’ and ‘PN40024’. Note additional gene family members (*PAL1-4* through *PAL1-12*) exist in ‘GREM4’ but were not found in ‘PN40024’ – these genes are therefore considered paralogous; It is likely these gene family members arose independently in ‘GREM4’. All genes are listed along with their expression. Expression is conveyed via horizontal bar graphs which illustrate expression via log2FoldChange where red is up-regulation and blue is down-regulation, compared to 0h controls. Green check marks indicate the change in expression was significantly different for the gene family member at the respective time point in question compared to 0h (significance was determined as p-adj ≤ 0.05 and a log2FoldChange ≥ 2). No DEGs were identified for ‘PN40024’ in the *PAL1* gene family in systemic leaves while four DEGs were identified in ‘GREM4’, some displaying temporally-specific expression.
